# Pulmonary Drug Delivery for Infectious Diseases: Cutting-Edge Formulations and Manufacturing Technologies

**DOI:** 10.3390/pharmaceutics18020242

**Published:** 2026-02-14

**Authors:** Brayan J. Anaya, Emanuel Osorio-Vargas, Samir Monterrosa-Moreno, Diego F. Tirado, Elena González-Burgos, Dolores R. Serrano

**Affiliations:** 1 Pharmaceutics and Food Technology Department, Faculty of Pharmacy, Universidad Complutense de Madrid, Plaza Ramón y Cajal s/n, 28040 Madrid, Spain; branaya@ucm.es; 2 Biological Engineering Program, Dirección Académica, Universidad Nacional de Colombia, Sede de La Paz, La Paz 202017, Colombia; dtiradoa@unal.edu.co; 3 Insumos y Equipos JR, Cartagena 130001, Colombia; emanuelosoriovargas@hotmail.com; 4 Facultad de ingeniería, Universidad de Cartagena, Cartagena 130002, Colombia; smonterrosam@unicartagena.edu.co; 5 Department of Pharmacology, Pharmacognosy and Botany, Faculty of Pharmacy, Universidad Complutense de Madrid, 28040 Madrid, Spain; 6 Instituto Universitario de Farmacia Industrial, Faculty of Pharmacy, Universidad Complutense de Madrid, 28040 Madrid, Spain

**Keywords:** pulmonary drug delivery, infectious respiratory diseases, aerodynamic properties, particle engineering, 3D printing use in lung therapy, lung-on-a-chip

## Abstract

Pulmonary drug delivery has emerged as a powerful strategy for the treatment of respiratory infectious diseases, including bacterial, fungal, and viral infections such as influenza and COVID-19, by enabling high local drug concentrations while minimizing systemic exposure. However, the clinical success of inhaled anti-infective therapies critically depends on the precise engineering of particle properties that govern lung deposition, cellular targeting, and therapeutic efficacy. In this review, we provide a comprehensive and technology-driven overview of cutting-edge formulation and manufacturing strategies for pulmonary drug delivery, with particular emphasis on the key process and formulation parameters required to generate effective inhalable systems for the treatment of infectious diseases. Advanced particle-engineering approaches, including spray drying, spray freeze drying, jet milling, and supercritical fluid technologies are discussed as enabling tools to tightly control aerodynamic particle size, morphology, and solid-state properties. In parallel, emerging platforms such as nanoparticle-based delivery systems are examined for their ability to target specific lung cell populations, including epithelial cells and alveolar macrophages, thereby enhancing antimicrobial efficacy. Finally, innovative manufacturing concepts such as microfluidics and three-dimensional (3D) printing are highlighted as promising strategies to improve particle size uniformity, reproducibility, and formulation customization. By integrating formulation science with advanced manufacturing technologies, this review identifies the critical design and processing parameters that underpin effective pulmonary delivery of anti-infective therapies and outlines future directions for the development of next-generation inhaled treatments.

## 1. Introduction

Pulmonary infectious diseases comprise a diverse spectrum of pathological conditions affecting the respiratory system, including the airways, alveoli, and pulmonary vasculature. These disorders are caused by various etiological agents, such as viral, bacterial, fungal, and parasitic pathogens, and contribute to significant morbidity and mortality worldwide [1]. These infections present significant challenges in diagnosis and treatment, particularly in the era of globalization and with the prevalence of chronic respiratory conditions.

Viral and bacterial lung infections are key factors in the pathogenesis and exacerbation of chronic respiratory diseases. Respiratory syncytial virus (RSV) and rhinoviruses are common triggers for asthma exacerbations and can influence the development of chronic respiratory diseases by inducing significant airway inflammation [2,3]. By June 2022, COVID-19 had caused over 6.3 million deaths globally, with notable regional variations in mortality rates. The global infection fatality rate was estimated at 0.15–0.20%, lower in individuals under 70 years of age. Excess mortality, reflecting deaths exceeding expected levels, revealed a global rate of 104.84 per 100,000 people, offering a broader view of the pandemic’s toll [4,5,6]. The COVID-19 pandemic has underscored the severe impact of viral infections on pulmonary health, with SARS-CoV-2 causing unprecedented morbidity and mortality. This global health crisis has emphasized the critical importance of molecular diagnostics and vaccination strategies in managing viral lung infections [7,8]. Patients with chronic lung diseases such as Chronic Obstructive Pulmonary Disease (COPD), asthma, and interstitial lung diseases (ILDs) are more vulnerable to respiratory infections, which can worsen their conditions and contribute to disease progression. Viral infections, in particular, play a role in the pathogenesis and acute exacerbations of these chronic respiratory diseases [9,10,11]. For lung cancer patients, infections can significantly complicate treatment outcomes, especially when immunotherapy is involved. Bacterial and viral infections can alter immune responses, potentially affecting the effectiveness and safety of cancer treatments [12,13,14].

Lung cancer and COPD are major contributors to the global health burden. Lung cancer continues to be the leading cause of cancer-related deaths globally, responsible for 18% of cancer fatalities in 2020 (nearly 20 million new cancer cases and approximately 10 million cancer-related deaths) [15]. Alarmingly, demographic projections suggest that the annual incidence of new cancer cases will escalate to 35 million by 2050, marking a 77% increase from 2022 levels [16]. At the same time, COPD affects an estimated 210 million people globally and is expected to become the third leading cause of death by 2030 [17,18].

Parasitic and fungal lung infections, though less common, present significant clinical challenges due to their potential for severe outcomes. Parasitic lung diseases can present as focal or cystic lesions, pleural effusions, or diffuse pulmonary infiltrates. With the increasing global mobility, these infections have emerged in non-endemic regions, requiring heightened vigilance among healthcare providers for timely diagnosis and intervention [19,20,21,22]. Beyond the well-known pathogens Aspergillus and Mucorales, other filamentous fungi such as *Scedosporium*, *Fusarium*, and *Penicillium* are gaining recognition as clinically significant. These fungi are associated with conditions, such as severe asthma with fungal sensitization and allergic bronchopulmonary mycosis. Fungal infections of the respiratory system contribute significantly to infectious disease-related mortality, with over 150 million severe cases and approximately 1.7 million deaths each year [23,24]. Chronic Pulmonary Aspergillosis (CPA), a serious condition that often arises as a complication of pulmonary tuberculosis, has a global prevalence of 3 million cases. CPA is a major long-term consequence of tuberculosis, particularly in regions with a high incidence of tuberculosis [25,26]. Pulmonary cryptococcosis, caused by *Cryptococcus* species, is another serious fungal infection that can resemble malignancy, often leading to diagnostic delays. This condition requires comprehensive diagnostic strategies, including advanced imaging techniques and specialized laboratory tests, along with extended antifungal treatment, especially in severe cases [27].

Coinfections with bacterial and fungal pathogens present a significant challenge in COVID-19 patients, with studies showing varying prevalence rates. For instance, a systematic review reported a 23.5% rate of fungal-bacterial coinfections among hospitalized COVID-19 patients [28,29], while another study found a pooled prevalence of 12.6% for fungal coinfections in COVID-19 cases [30]. Intensive Care Unit (ICU) patients are particularly at risk, with one study indicating that 20.54% of ICU patients with COVID-19 had confirmed bacterial and/or fungal coinfections [31,32]. These coinfections are linked to higher mortality rates, as a study showed a 54.6% mortality rate among COVID-19 patients with fungal coinfections [33]. The presence of coinfections is also associated with prolonged hospital stays and increased mortality [34]. The most common bacterial pathogens in COVID-19 coinfections include *Klebsiella pneumoniae*, *Acinetobacter* spp., and *Pseudomonas* spp., while *Candida* spp. and *Aspergillus* spp. are frequently implicated fungal pathogens [35,36]. Managing coinfections in COVID-19 patients is complicated by the presence of multidrug-resistant organisms, highlighting the need for careful antibiotic stewardship to prevent further resistance. Early identification and treatment of fungal pathogens are critical to preventing complications and improving patient outcomes [28,30].

This review uniquely integrates three perspectives often treated separately: (1) infection-specific pathophysiology that dictates formulation requirements (biofilm penetration, altered mucus rheology, surfactant dysfunction); (2) cutting-edge manufacturing technologies (3D printing, microfluidics, supercritical fluid processing) enabling next-generation anti-infective formulations; and (3) critical analysis of clinical trial outcomes informing rational development strategies. By bridging fundamental particle engineering with translational clinical data, we provide actionable insights for researchers developing inhaled therapies against respiratory pathogens.

### Comparative Limitations of Pulmonary Drug Delivery

While pulmonary drug delivery offers significant advantages for treating respiratory infections, including direct access to the site of infection, high local drug concentrations, and reduced systemic exposure, several inherent limitations must be considered relative to oral and injectable routes. A comprehensive understanding of these limitations is essential for rational therapeutic decision-making and for guiding future formulation development efforts [37,38].

Device technique dependency represents a primary challenge in pulmonary drug delivery. Effective inhalation therapy requires proper device handling, coordination between actuation and breathing, and adequate inspiratory flow rates. Multiple studies have demonstrated that 50%–80% of patients exhibit at least one critical error in inhaler technique, significantly compromising drug deposition and therapeutic outcomes. Common errors include failure to exhale before inhalation, inadequate breath-hold after inhalation, incorrect device positioning, and insufficient inspiratory flow for dry powder inhalers. These technique-dependent factors introduce substantial variability in delivered dose that is largely absent with oral medications, which require minimal patient technique, and injectable routes, which are typically administered by trained healthcare professionals. Patient education and regular technique reassessment are, therefore, essential components of inhaled therapy but add to the healthcare burden [39,40].

Cost considerations generally favor conventional delivery routes. Inhalation devices and their formulations are typically more expensive to develop and manufacture than oral solid dosage forms. The specialized engineering required for aerosol generation, including device components such as metering valves, actuators, and desiccants, increases production costs substantially. Additionally, inhaled products require specialized stability studies under various temperature and humidity conditions, device-formulation compatibility testing, and extensive in vitro aerodynamic characterization that add to development timelines and expenses. The complexity of manufacturing sterile nebulizer solutions or maintaining powder properties within narrow specifications further contributes to higher production costs compared to conventional tablets or capsules [41,42,43].

Drug delivery variability poses another significant limitation of the pulmonary route. Even with optimal inhalation technique, only 25%–40% of the emitted dose typically reaches the target lung regions, with the remainder depositing in the oropharynx, being exhaled, or remaining in the device. This lung deposition fraction shows considerable inter-patient variability influenced by factors including disease state (airway obstruction, mucus hypersecretion), breathing patterns (tidal volume, inspiratory flow rate), and individual airway geometry. Intra-patient variability also occurs due to day-to-day differences in disease severity and technique consistency. In contrast, oral bioavailability, while variable among drugs, tends to be more predictable for a given formulation, and injectable routes achieve near-complete systemic availability with minimal variability [44,45,46].

Formulation complexity requirements restrict the range of drugs suitable for pulmonary delivery. Active pharmaceutical ingredients must be formulated within narrow aerodynamic particle size distributions, typically 1 μm–5 μm mass median aerodynamic diameter for deep lung delivery to achieve effective deposition. The drug must maintain chemical stability during the aerosolization process, which may involve exposure to propellants, shear forces during nebulization, or mechanical stresses during dry powder dispersion. Furthermore, formulations must avoid causing local irritation, bronchoconstriction, or cough that would compromise patient adherence. Many drugs that are readily formulated for oral or parenteral administration cannot meet these stringent pulmonary requirements, limiting the applicability of this route [47,48,49].

Local adverse effects, including dysphonia (voice changes), oropharyngeal candidiasis (particularly with inhaled corticosteroids), cough, throat irritation, and bronchospasm, can occur with inhaled medications. These effects, while typically mild, can significantly impact patient adherence and quality of life [50,51]. Dry powder formulations may cause more throat irritation than nebulized solutions, as demonstrated with tobramycin inhalation powder versus solution. While systemic exposure is reduced compared to oral or parenteral administration, local toxicity to the respiratory epithelium, including potential effects on mucociliary clearance, surfactant function, and epithelial integrity, requires careful evaluation during product development [52].

Finally, regulatory complexity presents additional challenges for inhaled products. Pulmonary drug products face unique regulatory requirements, including demonstration of bioequivalence through complex pharmacokinetic studies (often requiring both systemic exposure and pulmonary deposition endpoints), pharmacodynamic equivalence studies, and comprehensive in vitro characterization of aerosol properties. Device-specific performance specifications, including delivered dose uniformity, fine particle fraction, and device robustness, add layers of testing not required for conventional dosage forms. The integrated nature of drug–device combination products means that changes to either component may necessitate bridging studies to demonstrate equivalence. These requirements add substantial time and cost to the approval process compared to conventional dosage forms [53,54].

## 2. Understanding the Physiology of the Respiratory System

**Figure 1:**
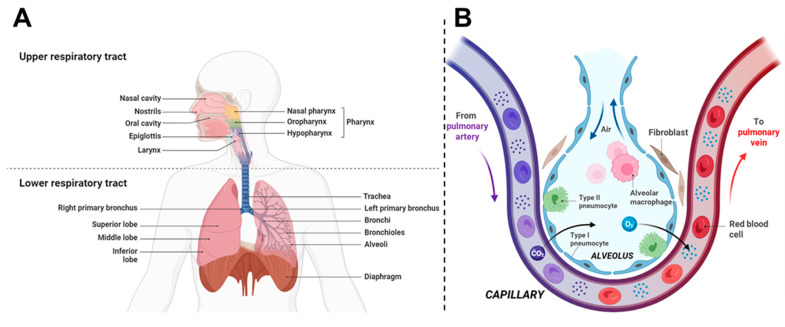
Anatomy and physiology of the respiratory system relevant to inhaled drug delivery. Key: (**A**) Regional organization showing the upper airways (nasal cavity, pharynx, larynx), tracheobronchial conducting zone (trachea through terminal bronchioles), and alveolar gas-exchange region (respiratory bronchioles through alveolar sacs) and (**B**) alveolar-interstitial region showing type I pneumocytes (gas exchange), type II pneumocytes (surfactant production), alveolar macrophages (innate defense), and the thin alveolar lining fluid layer. This region presents both a target and a barrier for inhaled antimicrobials.

The pulmonary system is a complex physiological network essential for gas exchange, acid-base homeostasis, and host defense. The respiratory tract is commonly segmented into the upper airways, the tracheobronchial (conducting) region, and the alveolar (gas-exchange) region, each with distinct epithelial types, mucus coverage, and cellular populations that determine both therapeutic targets and clearance pathways. The conducting airways are lined by ciliated pseudostratified epithelium and mucus that mediate mucociliary clearance, whereas the alveolar region is lined by thin type I and secretory type II pneumocytes involved in gas exchange and surfactant production, and is populated by alveolar macrophages that mediate particulate clearance and immune surveillance. The upper respiratory tract (nasal cavity, pharynx, larynx) serves as the first defense barrier, filtering particles larger than 10 μm through impaction. The tracheobronchial tree consists of 23 generations of branching airways with progressively decreasing diameters and increasing surface area. The alveolar region contains approximately 300 million–500 million alveoli in adult humans, providing the vast surface area essential for gas exchange and representing the primary target for deep lung drug delivery (Figure 1A) [55,56,57]. These anatomical structures work in concert to facilitate the passage and conditioning of inspired air, ensuring its filtration, humidification, and thermal equilibration before alveolar gas exchange.

The primary function of the respiratory system is to facilitate gas exchange, which primarily occurs at the alveolar-capillary interface of the lungs. This process involves the diffusion of oxygen into the bloodstream and the concurrent elimination of carbon dioxide, optimized by the larger alveolar surface area and a complex network of pulmonary capillaries [58]. However, the delicate structure of the lungs, while highly efficient for gas exchange, also makes them highly vulnerable to toxicants and pathogens, emphasizing the system’s vulnerability to various diseases and environmental threats [59].

The ventilatory mechanism, a key component of respiratory physiology, entails the rhythmic movement of air into and out of the lungs. This process is driven by the coordinated action of the diaphragm and intercostal muscles, which create pressure gradients within the thoracic cavity [60]. Additionally, the respiratory system is essential for maintaining acid-base balance by regulating the bicarbonate buffer system and adjusting ventilation rates in response to changes in blood carbon dioxide levels. Due to the respiratory system’s inherent susceptibility to external threats, ongoing advancements in medical technology and treatment strategies are critical for effectively addressing respiratory health challenges [61].

The cellular composition and function of the respiratory epithelium vary significantly across different regions of the lung. Club cells (formerly known as Clara cells) are primarily located in the bronchioles, where they play crucial roles in xenobiotic metabolism and surfactant protein secretion [62,63]. The alveolar-interstitial region (Figure 1B), which serves as the primary site for gas exchange, is predominantly lined by two distinct types of alveolar epithelial cells (AECs). Type I AECs, thin squamous cells covering approximately 95% of the alveolar surface, are essential for efficient gas exchange [64,65]. Type II AECs, cuboidal in shape and covering the remaining 5% of the surface, are responsible for pulmonary surfactant production and can differentiate into Type I AECs during injury repair processes [66].

Gas exchange occurs across the alveolar-capillary barrier, a structure comprising the alveolar epithelium, basement membrane, and capillary endothelium. This barrier is remarkably thin, approximately 0.5 μm in thickness, allowing for the efficient diffusion of gases [67]. Oxygen diffuses from the alveolar air space into the pulmonary capillaries, driven by the partial pressure gradient between alveolar gas and blood. On the other hand, carbon dioxide diffuses from blood into the alveoli to be exhaled. This process is governed by Fick’s law of diffusion and is influenced by factors such as the diffusion coefficient of the gas, the available surface area for exchange, and the thickness of the diffusion barrier [68,69].

The efficiency of gas exchange is further optimized by the ventilation-perfusion matching in the lungs, where regional blood flow is closely coordinated with regional ventilation to enhance gas exchange [70]. The interstitium, located between the alveolar epithelium and capillary endothelium, contains various cell types, including fibroblasts and immune cells, but is not typically considered to harbor epithelial cells [71]. This complex cellular architecture of the lung epithelium highlights its diverse roles in respiratory function, host defense, and tissue homeostasis. Recent advancements in single-cell sequencing technologies have deepened our understanding of lung cellular heterogeneity, revealing additional subpopulations with distinct molecular signatures and potential functional specificities [72]. A thorough understanding of alveolar gas exchange is crucial for comprehending various pathophysiological conditions, such as acute respiratory distress syndrome (ARDS), where disruption of the alveolar-capillary barrier leads to impaired gas exchange [73].

Understanding normal respiratory physiology establishes the baseline against which infection-induced alterations and their implications for antimicrobial delivery can be appreciated.

## 3. Progression of Infectious Diseases in the Respiratory Tract

Respiratory infections fundamentally alter the lung microenvironment described in Section 2. While healthy airways maintain a thin (2–5 μm) mucus layer with efficient mucociliary clearance (4–20 mm/min), bacterial infections induce mucus hypersecretion, increasing thickness to 50–200 μm with 10-fold–1000-fold elevated viscosity. The normally functional pulmonary surfactant becomes inactivated by plasma proteins and bacterial products, while biofilm formation creates additional diffusion barriers absent in healthy lungs. These pathological changes directly impact inhaled drug deposition, distribution, and therapeutic efficacy [74].

**Figure 2:**
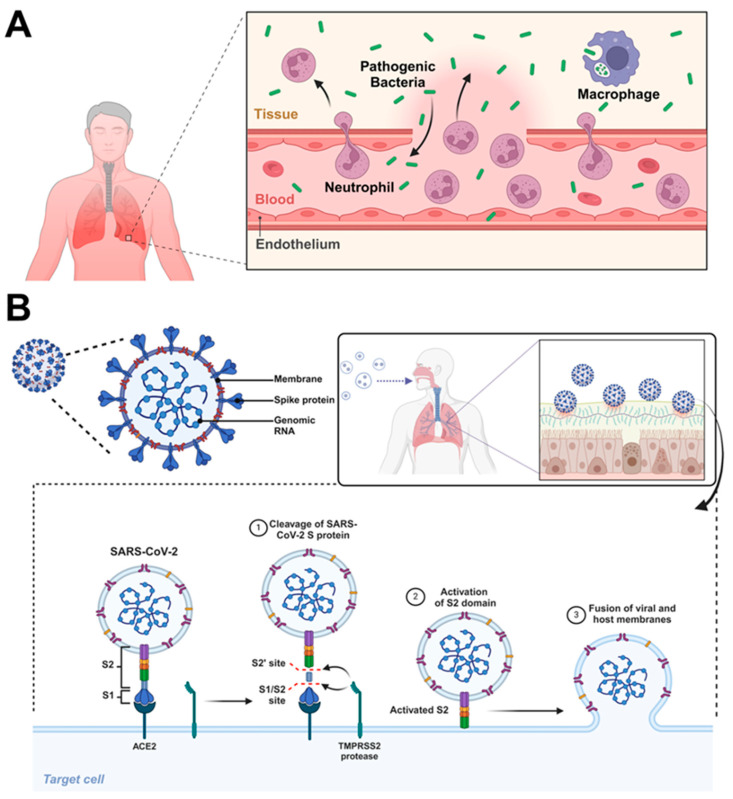
Pathophysiology of respiratory tract infections and implications for drug delivery. Key: (**A**) Bacterial infection showing mucosal invasion, inflammatory cell recruitment, mucus hypersecretion, and biofilm formation, and (**B**) viral infection demonstrating epithelial cell tropism, ciliary dysfunction, and inflammatory cascade.

The process of infection in the respiratory tract is a complex interaction between pathogens, host defense, and environmental factors (Figure 2). Respiratory infections can be caused by a wide range of microorganisms, including bacteria, viruses, and fungi, each with unique mechanisms of infection and pathogenesis. A comprehensive understanding of these processes is crucial for developing effective prevention and treatment strategies. This process can be described in several key stages:

(1) *Pathogen entry and colonization*: Pathogens generally enter the respiratory system by inhaling contaminated droplets or particles. The upper respiratory tract, including the nasal passages and pharynx, is often the initial site of colonization. In some cases, pathogens may also reach the respiratory system through hematogenous spread [75].
  Bacterial Infections: Pneumococcal bacteria, for example, employ specific ligand-receptor interactions to colonize the respiratory tract, invade the lungs, and potentially spread to the bloodstream and brain. This process involves a series of molecular events that can lead to severe disease symptoms if not controlled [76].
  Viral Infections: Viruses such as the influenza A virus, as well as emerging pathogens such as SARS-CoV-2 and H7N9, have distinct entry mechanisms. These viruses typically target the mucosal surfaces of the respiratory tract to initiate infection. The upper respiratory tract is particularly vulnerable, as it tends to have higher viral loads and faster infection resolution compared to the lower respiratory tract [77,78,79].
  Fungal Infections: The entry mechanism of fungal pathogens into the respiratory system involves several key steps. Fungal spores or conidia first evade the mucociliary clearance of the upper respiratory tract, reaching the lower airways and alveoli [80,81]. Once in the alveolar spaces, fungal spores can adhere to pulmonary epithelial cells through specific receptor-ligand interactions [82]. In individuals with compromised immune systems or impaired mucosal defenses, the immune response, primarily mediated by alveolar macrophages and neutrophils, may not be sufficient to clear the fungal elements effectively. Consequently, the spores can germinate into more invasive forms, such as hyphae or yeast, enabling penetration of the respiratory epithelial barrier [83]. This process facilitates tissue invasion and triggers localized inflammatory responses, eventually leading to clinical infection [84].
(2) *Overcoming Host Defenses*: The respiratory system has several innate defense mechanisms to prevent pathogen colonization, including (a) mucociliary clearance which is a mucus layer that traps particles and pathogens and then moved them upward by ciliary action, (b) antimicrobial peptides which are secreted by epithelial cells and possess broad-spectrum antimicrobial activity, and (c) resident alveolar macrophages that can phagocytose and eliminate invading pathogens. For pathogens to establish infection, they must overcome these defenses. This may involve strategies such as inhibition of ciliary function, degradation of antimicrobial peptides, or evading phagocytosis [66].
(3) *Adherence and Invasion*: The transmission of respiratory pathogens is strongly influenced by fluid dynamics, which govern the encapsulation, emission, and transport of pathogens in respiratory droplets [85]. Pathogens adhere to respiratory epithelial cells using various adhesins that bind to specific receptors on host cells. For example, influenza viruses bind to sialic acid residues, while *Streptococcus pneumoniae* uses surface proteins to attach to epithelial cells. After adherence, some pathogens may invade the epithelial cells directly or penetrate between them to reach underlying tissues [86]. Understanding these dynamics is essential for assessing transmission risks and developing effective control strategies.
(4) *Replication and Spread*: After establishing infection, pathogens begin to replicate within the host environment. Viruses hijack host cellular machinery for replication, while bacteria multiply using available nutrients. As the pathogen population grows, the infection can spread locally within the respiratory tract or, in some cases, systemically, affecting other organs or tissues [76].
(5) *Tissue Damage and Inflammation*: The presence and replication of pathogens stimulate host inflammatory responses, including the release of pro-inflammatory cytokines and chemokines that recruit immune cells to the site of infection. While this response is essential for pathogen clearance, it can also lead to tissue damage. For example, neutrophils release proteases and reactive oxygen species, which not only target pathogens but can also cause harm to host tissues, contributing to inflammation and damage [87].
(6) *Resolution or Progression*: The outcome of the infection depends on the balance between pathogen virulence and host defense mechanisms. In many cases, the immune response successfully clears the infection, resulting in recovery. However, if the pathogen overcomes host defenses or if the immune response is excessive, the infection may progress, potentially leading to severe conditions such as pneumonia or ARDS [88].
(7) *Tissue Repair and Remodeling*: After the infection is cleared, the respiratory system undergoes repair and remodeling processes. This involves the proliferation and differentiation of epithelial cells to restore the integrity of the respiratory epithelium. However, in some cases, especially following severe or recurrent infections, this repair process may result in long-term changes to respiratory function, potentially leading to chronic conditions or impairments in lung health [89].

These pathophysiological changes dictate specific requirements for inhalation devices used in anti-infective therapy.

## 4. Inhalation Devices

Pulmonary drug delivery is a promising approach for treating lung infections, offering direct access to lung tissues while minimizing systemic side effects. This approach is particularly advantageous for delivering antimicrobials and other therapeutics directly to the site of infection. Despite the potential of inhalable antimicrobial agents, only a few have received regulatory approval for clinical use, underscoring the need for ongoing research and development in this area [90,91,92]. Optimizing pulmonary drug delivery systems offers the opportunity to improve the effectiveness of treatments for various respiratory infections and chronic lung diseases.

**Figure 3:**
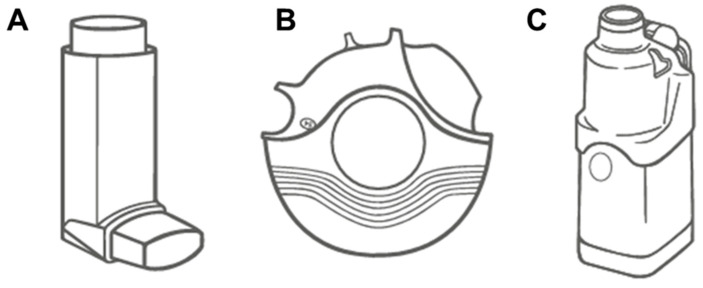
Commercially available inhalation devices for pulmonary drug delivery. Key: (**A**) Metered-dose inhaler showing propellant-driven aerosol generation through a metering valve—requires coordination between actuation and inhalation; (**B**) dry powder inhaler illustrating breath-actuated powder dispersion—requires adequate inspiratory flow (typically >30 L/min); (**C**) nebuliser demonstrating compressed air-driven aerosol generation from liquid formulations—longer treatment times but no coordination required. Adapted from [106].

The primary modes of pulmonary drug delivery include pressurized metered-dose inhalers (pMDIs), dry powder inhalers (DPIs), and nebulizers, each offering distinct advantages and limitations based on their design and patient needs [38,93,94]. pMDIs are widely used due to their portability and ease of use, delivering a precise dose of medication as a fine mist propelled by hydrofluoroalkane (HFA) propellants. Recent advancements in pMDI technology focus on optimizing particle size and velocity to enhance drug deposition patterns and minimize oropharyngeal deposition [95,96]. DPIs, which are propellant-free, have become increasingly popular for their ability to deliver higher doses. These devices rely on the patient’s inspiratory effort to disperse the drug. Innovations in DPI technology have led to the development of active DPIs, which use energy sources to aid in powder dispersion, improving dose consistency across different inspiratory flow rates [97,98]. Nebulizers convert liquid medications into fine aerosol droplets, commonly used in both hospital and home care settings. They are particularly effective in delivering high drug doses over extended periods, making them suitable for patients with acute respiratory infections or those unable to use other inhalers. Although vibrating mesh nebulizers offer superior performance, all nebulizer types face limitations, including bulky size, power requirements, and lengthy administration times. Recent technological advances include smart nebulizers that adapt to patient breathing patterns, enhancing delivery efficiency (Figure 3) [99,100].

Recent advances in inhalation technology have introduced soft mist inhalers (SMIs) and vibrating mesh nebulizers (VMNs) with significant advantages for anti-infective delivery. SMIs, exemplified by the Respimat^®^ device, generate slow-velocity aerosol clouds (~0.8 m/s vs. 2–8 m/s for pMDIs) through a spring-driven uniblock mechanism, achieving lung deposition of 40%–53% compared to 10%–25% for conventional pMDIs [101,102]. VMNs utilize piezoelectric elements vibrating at 100–180 kHz to generate aerosol through precision-engineered apertures, offering minimal residual volume (<0.1 mL), high fine particle fractions (>70%), and compatibility with viscous antibiotic formulations. A 2024 meta-analysis demonstrated superior clinical outcomes with VMNs versus jet nebulizers in respiratory patients [103]. Breath-actuated adaptive aerosol delivery systems (e.g., I-neb^®^, AKITA^®^) further optimize anti-infective delivery by synchronizing nebulization with inspiratory flow, achieving peripheral lung deposition of 70–80% [104,105].

Device selection must be matched with appropriately engineered particle properties to achieve effective antimicrobial deposition in infected airways.

## 5. Particle Engineering

Pulmonary physiological barriers that limit residence time and target access include mucociliary clearance in the conducting airways, phagocytosis by alveolar macrophages, epithelial tight junctions and transcellular transport limits, and the presence of lung surfactants that alter particle interfacial behavior and dispersion; overcoming or exploiting these barriers is central to inhaled particulate design. Specific clearance behaviors vary by region and disease state; for example, mucociliary clearance rapidly removes deposited material from large airways with clearance half-times of minutes to hours, while alveolar macrophage-mediated clearance operates on longer timescales (hours to days) and is a major sink for particulates reaching the deep lung [57,107,108].

The efficacy of inhaled therapeutics for treating pulmonary infections is intrinsically linked to particle engineering, which integrates principles of aerosol science, materials engineering, and pharmaceutical technology. Different pathogens colonize distinct anatomical sites within the respiratory tract, necessitating tailored aerodynamic properties for effective drug delivery. For instance, the use of large porous particles and nanoparticle aggregates has shown promise in enhancing deep lung deposition and sustained drug release [109]. These approaches aim to overcome physiological barriers and improve therapeutic outcomes for various pulmonary and systemic diseases.

**Figure 4:**
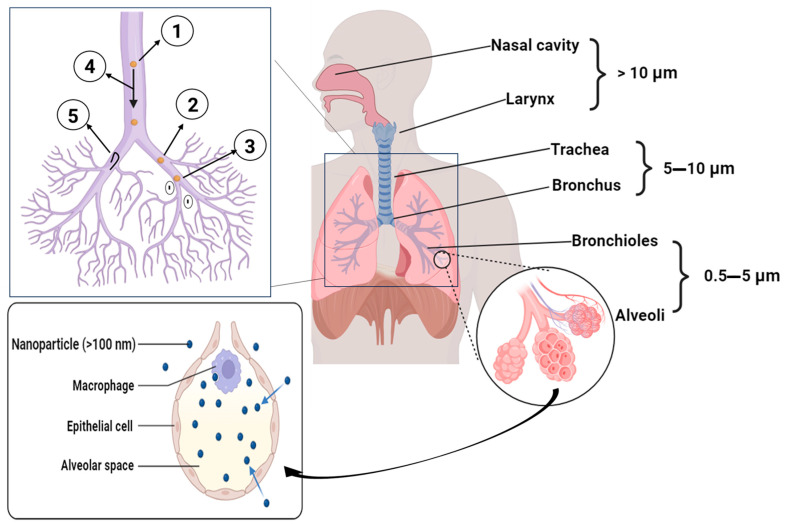
Particle deposition mechanisms in the respiratory tract. Keys: (**1**) impaction—large (particles with high momentum cannot follow airstream directional changes at bifurcations, depositing on airway walls, predominant for particles >5 μm in upper airways), (**2**) sedimentation—medium (particles settle under gravity during breath-holding or slow breathing, predominant for 1–5 μm particles in small airways and alveoli), (**3**) electrostatic deposition, (**4**) particle trajectory, and (**5**) diffusion—small (≤0.1 μm) (random motion causes submicron particles to contact airway walls, significant only for nanoparticles). The optimal aerodynamic diameter for lower respiratory tract deposition of antimicrobials is 1–5 μm.

Particle deposition in the respiratory tract is dominated by three physical mechanisms whose importance depends principally on aerodynamic particle diameter (Da) including inertial impaction that predominates for larger particles (typically Da ≳ 5 μm) and governs deposition in the upper airways and oropharynx, gravitational sedimentation that is important in the 1–5 μm range and favors tracheobronchial and peripheral deposition, and Brownian diffusion that dominates the deposition of very small particles (typically Da ≲ 0.5–1 μm) and enhances alveolar deposition for nanoparticles and ultrafine aerosols (Figure 4). For practical inhalation products, mass median aerodynamic diameters (MMAD) in the ~1–5 μm window are usually targeted to achieve significant peripheral lung deposition, with formulations often engineered to produce respirable fractions below 5 μm and MMAD values near ~1–3 μm for deep lung delivery [110,111].

The Da differs from geometric diameter (Dg) and is defined as the diameter of a unit-density sphere with the same settling velocity as the particle in question:

(1) $$Da=Dg\sqrt{\frac{\rho p}{\rho 0}}$$

where ρp is particle density, and ρ0 is unit density (1 g/cm^3^). This relationship explains why large porous particles with low density can exhibit small aerodynamic diameters suitable for deep lung penetration despite large geometric sizes [112]. While Dg describes the actual physical size of a particle, Da predicts its deposition behavior. This distinction enables the design of large porous particles with low aerodynamic diameters that combine good flow properties and aerosolization characteristics with deep lung penetration.

Equation (1) derives from Stokes’ law describing particle settling in viscous media, formalized for aerosol science by Fuchs. The aerodynamic diameter concept enables comparison of particles with different densities and shapes by expressing their behavior as equivalent unit-density spheres [110,111,113].

Particles smaller than 1 μm, particularly those in the nanoscale range, are generally exhaled. However, a subset of these particles can penetrate the alveolar region through Brownian diffusion [114,115]. The deposition of particles in the respiratory system is intricately linked to aerodynamic particle size, involving particle size and density, influenced by multiple interacting factors. Particles between 0.1 and 1 μm have the highest probability of reaching the alveoli, as they are small enough to avoid upper airway filtration but large enough to settle in the deep lung. Particles smaller than 0.5 μm primarily deposit via Brownian diffusion, while larger ones are more significantly affected by inertial impaction and gravitational settling [116,117,118,119,120]. This size-dependent behavior has been leveraged in pharmaceutical research, leading to the development of innovative drug delivery systems such as the “Trojan horse” approach. In this strategy, nanoparticles are encapsulated within larger microparticles, combining the advantages of efficient aerosolization with deep lung penetration and enhanced alveolar absorption, ultimately optimizing drug delivery to the deep lung [121,122,123,124,125,126,127].

Particle deposition mechanisms in the respiratory tract. (A) Inertial impaction: particles with high momentum cannot follow airstream directional changes at bifurcations, depositing on airway walls; predominant for particles >5 μm in the upper airways. (B) Gravitational sedimentation: particles settle under gravity during breath-holding or slow breathing; predominant for 1–5 μm particles in small airways and alveoli. (C) Brownian diffusion: random motion causes submicron particles (<0.5 μm) to contact airway walls; significant only for nanoparticles. The optimal aerodynamic diameter for lower respiratory tract deposition of antimicrobials is 1–5 μm.

In general, MMAD, fine particle fraction (FPF), and geometric standard deviation (GSD) are some fundamental parameters in aerosol science by mapping the aerodynamic particle size distribution (APSD) to specific anatomical regions based on physical deposition mechanisms [128]. By analyzing these three metrics, scientists can use models and in vitro data from devices such as cascade impactors to quantitatively predict the mass of drug delivered to and deposited in specific lung regions.

MMAD represents the aerodynamic diameter at which 50% of the total mass of aerosolized particles is composed of particles smaller than this value, and 50% is composed of particles larger. As the median of the particle mass distribution, the MMAD is a key indicator of particle deposition within the respiratory tract. Smaller MMAD values typically result in deposition in the peripheral airways (alveoli), whereas larger MMAD values lead to deposition in the central airways. MMAD is determined by factors such as particle density, aerodynamic properties, and the measurement method used, all of which can influence its accuracy and relevance in various inhalation applications [92,129,130]. Two general approaches are commonly used for its determination, depending on the type and availability of cascade impactor data.

When individual stage data are available, a mass-weighted geometric mean of the aerodynamic stage cut-off diameters can be calculated using Equation (2):

(2) $$MMAD=exp\left(\frac{\sum_{i=1}^{n}m_{i}ln(d_{a,i})}{\sum_{i=1}^{n}m_{i}}\right)$$

where $m_{i}$ is the mass of drug collected on stage i, $d_{a,i}$ is the corresponding aerodynamic cut-off diameter, and n is the number of stages in the impactor. This expression provides a mass-weighted mean aerodynamic diameter, assuming a log-normal distribution of particle sizes, and is frequently used for comparative or modeling purposes when complete cumulative data are not available.

Alternatively, the pharmacopeial and experimentally preferred method defines MMAD as the aerodynamic diameter corresponding to the 50th percentile of the cumulative mass distribution. It is obtained by logarithmic interpolation between the two consecutive stages that bracket the 50% cumulative mass point, using Equation (3):

(3) $$\log_{10}\left(MMAD\right)=\log_{10}\left(D_{p,1}\right)+\frac{\left(50-F_{1}\right)\left({log}_{10}(D_{p,2})-{log}_{10}(D_{p,1})\right)}{F_{2}-\;F_{1}}$$

where $D_{p,1}$ and $D_{p,2}$ are the aerodynamic diameters of the lower and upper stages surrounding the 50% cumulative mass fraction, and $F_{1}$ and $F_{2}$ are the corresponding cumulative mass percentages. This logarithmic interpolation method assumes that aerosol particle size distributions are log-normally distributed, which is typical for inhalation aerosols. The log-probability plot (log of aerodynamic diameter vs. cumulative percent undersize) is approximately linear, allowing accurate determination of MMAD at the 50% intercept [131].

FPF refers to the percentage of particles in an aerosol with an aerodynamic diameter of less than 5 μm, which is critical for assessing drug delivery to the deep lung regions, particularly the alveoli, where gas exchange occurs [132]. The FPF is calculated using Equation (4):

(4) $$FPF\;\left(\%\right)=\frac{Mass\;of\;particles<5\;\mu m}{Total\;mass\;of\;inhaled\;particles\;}\;\times 100$$

where the mass of particles < 5 µm is the mass of particles with an aerodynamic diameter less than 5 μm, typically measured using a cascade or next-generation impactor. The total mass of inhaled particles is the overall mass of aerosolized particles. FPF is expressed as a percentage and represents the fraction of the dose expected to reach the lower respiratory tract [92,133]. The MMAD is closely related to FPF. Smaller MMADs generally result in higher FPFs, which enhance lung deposition. For example, particles with an MMAD of around 1.5 µm have been shown to achieve significant lung deposition, with FPFs being a critical determinant of this outcome [134].

Equations (2)–(4) represent pharmacopeial standards for cascade impactor analysis. MMAD is determined from cumulative mass distributions, while FPF quantifies the respirable fraction (<5 μm) capable of reaching lower airways—a critical quality attribute for inhaled anti-infectives [135].

The GSD measures the distribution or variability of particle sizes within an aerosol. A lower GSD indicates that the aerosol is more monodisperse, meaning the particles are similar in size, whereas a higher GSD suggests a polydisperse aerosol, where particle size varies significantly. GSD is an important parameter for assessing the consistency of particle sizes in inhalation formulations [129,130,136]. The GSD is calculated using Equation (5):

(5) $$GSD=exp\left(\sqrt{\frac{\sum (\ln D_{a,i}-\ln MMAD{)}^{2}\;}{N-1}}\right)$$

where *N* is the number of particle size stages or data points, MMAD is the mass median aerodynamic diameter, and *D_a_*_,*i*_ represents the aerodynamic diameter of the *i*-th particle.

The aerodynamic diameter *D_a_*_,*I*_ of the *i*-th particle is defined as the diameter of a unit-density sphere (1 g/cm^3^) that exhibits the same aerodynamic behavior, such as velocity and settling characteristics, as the actual particle. This measurement considers not only the particle’s physical size but also its density and shape. Thus, the aerodynamic diameter reflects the particle’s effective diameter in an airstream, rather than its true geometric size. By accounting for variations in shape and density, the aerodynamic diameter enables the comparison of particles with different compositions, providing a standardized measure of how particles behave in airflow and deposit within the respiratory tract [137,138,139].

Alternatively, the GSD can be approximated from the ratio of the diameters corresponding to 84.1% and 50% (for the upper bound) or 50% and 15.9% (for the lower bound) of the cumulative mass distribution curve. This is given by Equation (6) [92]:

(6) $$GSD=\;\frac{D_{a,84.1\%}}{D_{a,50\%}}=\;\frac{D_{a,50\%}}{D_{a,15.9\%}}$$

where *D_a_*_,84.1%_ is the aerodynamic diameter at which 84.1% of the particle mass is smaller, *D_a_*_,50%_ is the MMAD, *D_a_*_,15.9%_ is the aerodynamic diameter at which 15.9% of the particle mass is smaller. In log-normal distributions, the GSD characterizes the breadth of the particle size distribution, indicating how wide or narrow the particle size range is.

For pulmonary drug delivery, GSD values between 1.5 and 2.5 are desirable, balancing manufacturing feasibility with aerosol performance. Highly monodisperse aerosols (GSD < 1.5) often exhibit poor powder flow and aerosolization characteristics despite theoretical advantages.

Equations (5) and (6) describe GSD, characterizing aerosol polydispersity based on log-normal distributions first rigorously described by Raabe (1971) [140]. GSD values < 2.0 indicate monodisperse aerosols preferred for consistent antimicrobial deposition.

For bacterial infections in cystic fibrosis, *P. aeruginosa* predominantly colonizes the bronchioles and bronchi, requiring particles with an MMAD of 2–5 μm for optimal deposition in these conducting airways [90,92]. Studies using radiolabeled aerosols have demonstrated that particles with MMAD of 3.0–3.5 μm achieve 40–60% deposition in bronchial regions where bacterial biofilms form [141]. For community-acquired pneumonia affecting the alveolar spaces, particles with MMAD < 3 μm are essential to reach the gas-exchange regions where *Streptococcus pneumoniae* and other pathogens establish infection [76].

Invasive pulmonary aspergillosis caused by *Aspergillus fumigatus* primarily affects the alveolar regions and small airways, requiring particles with an MMAD of 1–3 μm for adequate lung penetration [142]. Computational fluid dynamics (CFD) modeling has shown that particles with MMAD of 2.5 μm achieve 35–45% alveolar deposition, which is optimal for delivering antifungal agents to sites of fungal colonization [143,144]. For allergic bronchopulmonary aspergillosis (ABPA), which affects larger airways, slightly larger particles (MMAD 3–5 μm) are more appropriate [145,146].

Respiratory viruses such as SARS-CoV-2 and influenza initially infect the upper respiratory tract but can progress to involve the entire tracheobronchial tree and alveoli. A bimodal particle size distribution combining larger particles (MMAD 4–6 μm) for upper airway deposition and smaller particles (MMAD 1–3 μm) for alveolar delivery may provide comprehensive coverage for antiviral therapy [134,147].

Engineered aerosol properties require rigorous characterization to ensure consistent anti-infective delivery.

## 6. Assessment of Pulmonary Deposition

**Figure 5:**
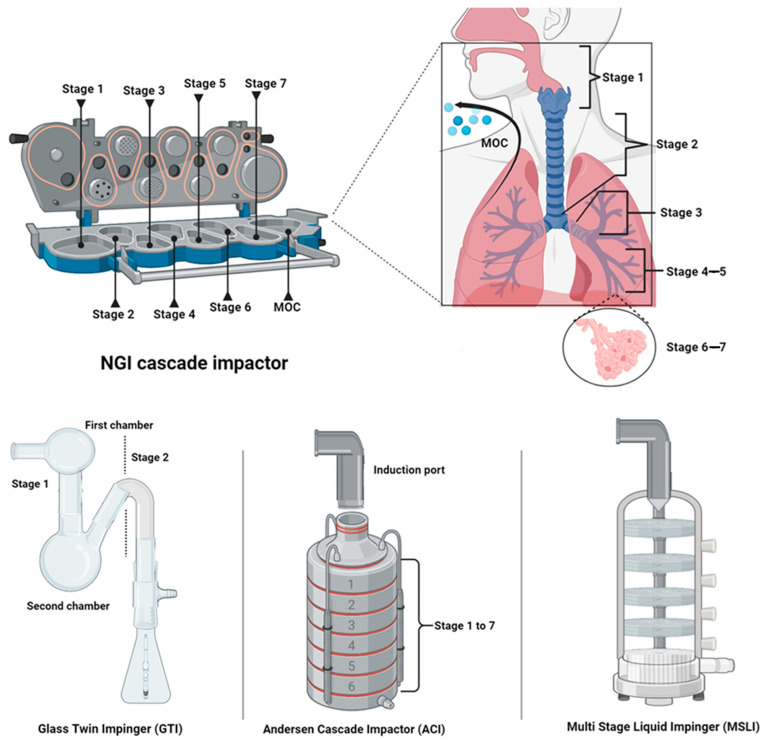
In vitro devices for characterizing aerosol particle size distribution. Keys: Next Generation Impactor (NGI): 7-stage impactor with pre-separator; designed for higher flow rates and improved stage efficiency. Glass Twin Impinger (GTI): simplified 2-stage apparatus separating respirable (<6.4 μm) from non-respirable fractions. Andersen Cascade Impactor (ACI): 8-stage impactor with progressively decreasing cutoff diameters; pharmacopeial standard. Multi-Stage Liquid Impinger (MSLI) consists of 4 impactor stages plus a final filter stage (5 collection stages total). Designed for dry powder inhaler (DPI) testing, it employs liquid in each stage to prevent particle bounce.

Measuring pulmonary deposition is crucial in inhalation drug delivery to ensure that aerosolized medications reach the intended lung regions for optimal therapeutic effects. The Next Generation Impactor (NGI) is one of the most widely used devices for determining the APSD and predicting deposition in different regions of the respiratory tract (Table 1) [148,149,150]. However, other instruments and methods are also available for assessing lung deposition (Table 2 and Figure 5). For instance, the Andersen Cascade Impactor (ACI) and Multi-Stage Liquid Impinger (MSLI) are often used due to their ability to simulate aerodynamic particle behavior and regional lung deposition. Simpler devices like the Glass Twin Impinger (GTI), Fast Screening Andersen (FSA), and Fast Screening Impactor (FSI) are useful during early-stage development for providing quick, lower-resolution assessments [148,151,152,153,154,155,156]. While laser diffraction is not typically employed to directly measure lung deposition, it is used to assess particle size distribution (PSD) in aerosol formulations. This is an important factor in determining how particles behave within the respiratory system. By analyzing the PSD, laser diffraction can provide indirect insights into how particles may deposit in different lung regions based on their aerodynamic behavior [157,158,159]. The choice of method depends on the stage of drug development, required resolution, and the complexity of the inhaled formulation.

*NGI* is a multi-stage cascade impactor designed to measure the aerodynamic particle size distribution of inhaled aerosols. It operates by separating particles based on their aerodynamic diameters, ranging from large particles that tend to deposit in the upper airways to smaller ones that can penetrate deeper into the lungs. Each stage of the NGI represents a distinct region of the respiratory tract, allowing for an accurate estimation of where particles are likely to deposit within the lungs [148].

The NGI typically consists of seven stages plus a micro-orifice collector (MOC). Each stage has a specific cut-off diameter, which represents the aerodynamic diameter at which particles have a 50% probability of impaction. These cut-off diameters are flow-rate dependent [150,160,161]. For a standard flow rate of 60 L/min, the cut-off diameters are as follows: 8.06 µm, 4.46 µm, 2.82 µm, 1.66 µm, 0.94 µm, 0.55 µm, 0.34 µm from stage 1 to stage 7, and <0.34 µm to MOC, respectively (Table 1) [162].

Validated assessment methods guide manufacturing process optimization for inhaled antimicrobial products.

**Table 1:** The stage of the NGI correlates with specific regions of the lungs, based on aerodynamic diameter. These cut-off diameters are flow-rate dependent. Values reported in this Tble corresponds to a standard flow rate of 60 L/min.

L/min.Stage of the NGI	Aerodynamic Particle Size Range	Lung Region	Ref
Stage 1: Oropharyngeal region	>8 μm	Particles collected in Stage 1 are generally too large to penetrate the lungs and instead are deposited in the oropharyngeal and upper airway regions (mouth and throat). These particles do not contribute to therapeutic lung delivery but can cause local side effects such as irritation or an unpleasant taste.	[163,164,165]
Stage 2: Upper Tracheobronchial region	>5–8 μm	These particles primarily deposit in the upper tracheobronchial tree, which includes the large bronchi. Particles in this range may be cleared by mucociliary action and do not reach the lung.	[143,166,167]
Stage 3: Lower Tracheobronchial region	3–5 μm	Particles collected in Stage 3 are more likely to deposit in the lower tracheobronchial tree, specifically in the smaller bronchi and bronchioles. This is an important site for drugs treating bronchoconstriction and airway inflammation, such as bronchodilators and corticosteroids.	[166,167,168,169]
Stage 4–5: Bronchiolar region	1–3 μm	These particles represent deposition in the terminal bronchioles, which are critical for respiratory diseases such as asthma and COPD. Effective deposition in this region is necessary for medications aimed at reducing airway inflammation and preventing bronchoconstriction. Also, particles in this range are small enough to reach the alveolar ducts. Drugs deposited in this region are crucial for treating diseases like pulmonary hypertension or delivering systemic therapies that require deep lung absorption.	[143,170,171]
Stage 6–7: Alveolar region	<1 μm	Particles collected in Stage 6 typically deposit in the alveolar sacs. This region is responsible for gas exchange, making it a target for systemic drug delivery via the lungs. Effective deposition here is crucial for drugs that rely on absorption into the bloodstream, such as insulin. Stage 7 represents the deposition of fine particles in the deep alveolar region, close to the pulmonary capillaries. This region is key for highly efficient drug absorption due to the large surface area and proximity to the blood supply, which is essential for systemic treatments administered via the lungs.	[172,173]
MOC: Exhalation and/or nanoparticles deposition	<0.34 μm	Particles with an MMAD below 100 nm exhibit low inertia and are prone to exhalation before deposition occurs. However, nanoparticles can penetrate deeply into the alveolar region and even cross into the bloodstream. This is the focus for drug delivery systems involving nanotechnology and for inhalation toxicology studies involving ultrafine particles.	[174,175,176,177]

**Table 2:** Techniques to evaluate drug lung deposition. Key: NGI: Next Generation Impactor, GTI: Glass Twin Impinger, ACI: Andersen Cascade Impactor, MSLI: Multi-Stage Liquid Impinger, and FSI: Fast Screening Impactor.

Technique	Principle	Advantages	Limitations	Ref
**NGI**	It is a multi-stage cascade impactor that separates aerosol particles by aerodynamic diameter across several stages. Each stage corresponds to different lung regions, from large particles depositing in the upper airways to smaller particles reaching deep into the lungs.	High resolution across a wide range of particle sizes, making it highly accurate. Considered a regulatory standard for measuring FPF in inhalation aerosols. Provides detailed information on regional lung deposition.	Time-consuming setup, operation, and maintenance. Requires careful cleaning to prevent sample cross-contamination. High operational complexity and cost.	[150,178,179]
**GTI**	Consists of a simple, two-stage device designed to mimic the upper and lower regions of the respiratory tract. Aerosols pass through the stages, with larger particles depositing in the first stage (representing the oropharynx and upper airways), and smaller particles depositing in the second stage (representing the lower airways). The GTI separates particles based on their aerodynamic size, using liquid media in each stage to capture particles for analysis.	The GTI is cost-effective, easy to use, and offers a straightforward means to estimate deposition in the upper and lower respiratory tract. It provides useful data for screening DPIs and MDIs early in inhaled drug development.	Its primary limitation is the low resolution, as it only offers two stages of particle separation. This limits the granularity of data compared to other impactors, making it insufficient for detailed analysis of APSD across all regions of the lung.	[152]
**ACI**	This is a multi-stage device used to measure the APSD of aerosols. It separates particles by their aerodynamic diameter as the aerosol passes through a series of nozzles, with larger particles collecting in earlier stages and finer particles in later stages. Each stage simulates different regions of the respiratory tract, from the upper airways to the alveoli, allowing for detailed characterization of particle deposition.	The ACI provides high-resolution particle size distribution data across a wide range of particle sizes. It is considered the gold standard for in vitro aerosol testing, widely used in regulatory submissions due to its precision and reproducibility.	Despite its accuracy, the ACI is complex to set up and requires significant time and effort for testing, cleaning, and maintenance. Additionally, the manual nature of particle collection and stage handling increases the risk of sample loss or contamination between tests.	[180]
**MSLI**	Improves upon the GTI by providing additional stages, typically five, to separate particles by aerodynamic size with greater resolution. As the aerosol passes through each stage, particles deposit in liquid impingers according to their size, mimicking the progressive deposition in the respiratory tract.	The MSLI offers better resolution than the GTI, allowing for a more detailed analysis of the APSD. The use of liquid impingers reduces particle re-entrainment, improving the accuracy of measurements. It is particularly useful for characterizing the FPF of DPIs and MDIs.	While the MSLI improves on the GTI, it still lacks the high resolution of cascade impactors such as the ACI or NGI. It may not be as effective for characterizing particles at the submicron level, making it less suitable for detailed regulatory submissions.	[153,181,182]
**FSI**	This is a simplified version of a cascade impactor designed for rapid screening of APSD in aerosol formulations. It typically consists of a few stages, often two or three, to quickly classify aerosols into larger and smaller particle fractions. The FSI is particularly useful in early-stage formulation development, where rapid, high-throughput screening is required.	The FSI provides a fast and efficient method for assessing APSD, making it ideal for early-stage development where multiple formulations need to be tested in a short time. It requires minimal setup and cleaning compared to more complex impactors like the ACI or NGI.	The primary limitation of the FSI is its lower resolution compared to full cascade impactors. It provides limited data on particle size distribution and is not suitable for detailed regulatory submissions. Its use is primarily for screening purposes rather than in-depth analysis.	[183,184]

## 7. Manufacturing Technologies for Pulmonary Drug Delivery

**Figure 6:**
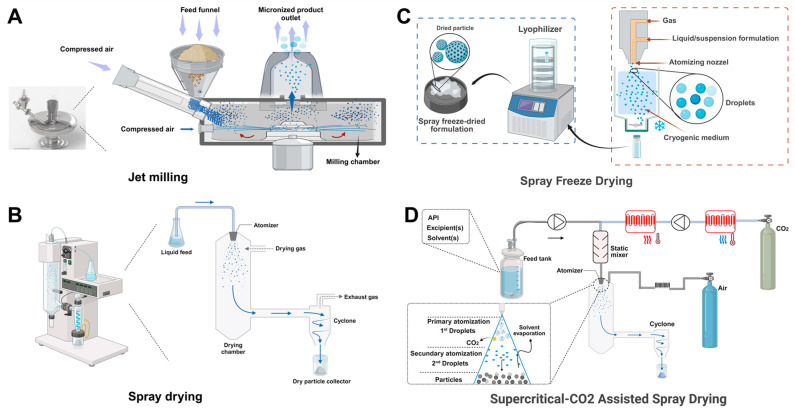
Manufacturing technologies for dry powder inhalation formulations. Key: (**A**) jet milling: high-energy particle size reduction producing micronized powders (typically 1–10 μm)—may induce amorphous content and electrostatic charge; (**B**) spray drying: atomization of liquid feed into heated chamber producing engineered particles with controlled size, morphology, and solid-state properties—widely used for antibiotics and carrier systems; (**C**) spray freeze drying: atomization into cryogenic medium followed by lyophilization—produces highly porous particles with excellent aerodynamic properties for deep lung delivery; (**D**) supercritical-CO_2_-assisted spray drying: CO_2_-based particle formation enabling solvent-free processing—particularly suitable for thermolabile compounds.

Various advanced manufacturing techniques are employed to produce inhalable particles with specific aerodynamic properties tailored for treating pulmonary infections. The selection of manufacturing methods significantly influences particle size distribution, morphology, density, and surface properties, ultimately affecting therapeutic efficacy and regional lung deposition. For pulmonary infections, achieving optimal particle deposition in infected lung regions is critical. Bacterial infections in bronchioles (such as in cystic fibrosis) require MMAD of 2–5 μm, while fungal infections in alveoli necessitate MMAD of 1–3 μm for adequate penetration. The main approaches include spray drying, lyophilization (freeze-drying), supercritical fluid technology, and jet milling, each characterized by unique mechanisms and specific advantages and limitations (Table 3 & Figure 6) [185,186,187].

Jet milling represents a crucial mechanical technique for particle size reduction, especially effective for crystalline drugs with inherent stability issues during spray drying or lyophilization, and utilized in the production of DPI formulations. For producing optimal dry powder formulations for lung deposition via jet milling, the essential parameters can be categorized into material attributes of the feed powder and critical process parameters of the jet mill operation [188]. The process employs high-velocity gas streams (typically nitrogen or air at 4–8 bar) to create particle-particle and particle-wall collisions, resulting in micronization to 1–5 μm particle sizes (Figure 6A) [186,189]. While jet milling produces particles of the correct size range, the inherent cohesion of the resulting powders can limit the delivered FPF. Formulation strategies, such as co-milling with force control agents (e.g., L-leucine) or formulating with coarse carrier particles (e.g., lactose blends), are often necessary post-milling to mitigate cohesive forces and enhance powder dispersibility, thereby optimizing the FPF and overall delivery efficiency. Optimized jet-milled formulations can achieve high FPF values [190,191]. For example, heparin sodium microparticles produced by co-jet-milling with leucine achieve an MMAD of 2.6–2.9 μm with a FPF of 61–76%, demonstrating effective particle size [192]. Jet milling operates entirely in the solid state, avoiding solvent-related complications and preserving crystalline structures essential for drug stability [193]. Modern jet mills, often integrated with real-time particle size analysis using laser diffraction, allow for precise control over particle size distribution [194]. Despite its advantages, jet milling poses challenges, including potential modifications to crystal structure through mechanical stress-induced amorphization, changes in surface energy that can impact powder flowability and aerosolization, and the generation of electrostatic charges causing particle aggregation. Additionally, the process requires careful optimization to achieve narrow particle size distributions (GSD < 2.0) suitable for consistent pulmonary delivery, as broader distributions result in variable lung deposition patterns [195].

Spray drying is a widely used method for generating inhalable particles, offering precise control of particle properties by adjusting various process parameters. The process involves atomizing a drug solution or suspension into fine droplets, followed by rapid solvent evaporation in a heated drying chamber (Figure 6B). Bearing in mind critical parameters, the inlet temperature governs particle morphology and drug stability such as temperatures above 120 °C which may create hollow, low-density particles (0.2–0.4 g/cm^3^) with favorable aerodynamic properties due to increased geometric diameter while maintaining optimal aerodynamic diameter, resulting in MMAD values of 2–3 μm and FPF > 60% [196,197,198,199]. However, high temperatures risk thermal degradation of heat-sensitive antibiotics and antifungals. Conversely, temperatures below 100 °C produce denser particles (0.6–1.0 g/cm^3^) with reduced dispersibility (FPF typically 30–45%) yet improved drug stability, particularly important for maintaining the activity of β-lactam antibiotics and polyene antifungals such as amphotericin B [200]. Feed concentration affects particle size and porosity; for example, dilute solutions (0.5–2% *w*/*v*) yield smaller, more porous particles (MMAD 1–3 μm) with enhanced aerosolization and FPF values of 65–80%, ideal for deep lung targeting in fungal infections. Concentrated feeds (>5% *w*/*v*) produce larger, denser particles (MMAD 4–6 μm) with poor aerodynamic performance (FPF < 40%) but higher drug loading, suitable for upper airway deposition in bacterial bronchitis [201]. Atomization pressure and airflow rate determine droplet size distribution and residence time. Higher atomization pressure increases the velocity and energy of the spray, leading to finer droplets. Increasing air pressure from 2 bars to 4 bars can decrease particle size from about 20 μm to 5 μm. Higher airflow rates can enhance the atomization process by providing more shear force, but if the airflow is too low, it can result in larger droplets, and if it is too high, it can cause issues like poor burning or excessive spray loss. Airflow rates of 400–800 L/min ensure adequate drying while preventing particle aggregation [202,203,204]. This technique has been successfully applied in commercial products such as Tobi Podhaler^®^ (tobramycin inhalation powder) for cystic fibrosis treatment. The particle size in median geometric diameter is around 1.7–2.7 μm [205,206,207,208], which could achieve FPF around of 37–68%, enabling effective delivery to airways colonized by *Pseudomonas aeruginosa* [209,210]. Recent advancements in spray drying technology have facilitated the development of composite particles. For instance, leucine-containing formulations create hydrophobic particle surfaces that reduce moisture uptake and improve powder dispersibility, increasing the FPF [211]. Mannitol-based carriers enable the production of porous particles with aerodynamic diameters suitable for alveolar deposition (MMAD 1.5–3 μm) while maintaining sufficient geometric size (≤5 μm) to avoid exhalation [212].

Freeze drying (lyophilization) is an established technology in pharmaceuticals, primarily for stabilizing sensitive products (e.g., proteins, vaccines) in vials. At the same time, traditional bulk or vial freeze drying is not a primary manufacturing method for respirable dry powders because it typically yields a non-flowable “cake” that requires intense post-processing (milling, sieving) to create inhalable particles. A specialized adaptation known as spray freeze drying (SFD) is used for engineering highly dispersible pulmonary particles.

SFD is an engineered approach where the formulation is atomized into droplets (like in spray drying) and immediately frozen in a cryogen medium (e.g., liquid nitrogen) before lyophilization. This bypasses the bulk cake formation issue and creates highly porous, spherical particles ideal for inhalation (Figure 6C) [213]. Atomization represents the initial and most crucial stage, in which the feed solution or suspension is disintegrated into fine droplets by an atomizer, a process that governs both the drying kinetics and the resulting particle size distribution. The selection of a suitable atomizer is therefore essential, as it directly influences both process efficiency and economic viability, while also shaping the morphological and physical properties of the final particles. Different atomizer types offer distinct performance characteristics [214]. Hydraulic (pressure) atomizers, producing droplets of approximately 120–250 µm, yield powders of higher density and excellent flowability, although they may generate less homogeneous particles and are susceptible to corrosion. Pneumatic (two- or four-fluid) atomizers produce finer droplets (5–100 µm) and offer superior particle size control, suitable for viscous formulations, but their high compressed gas consumption increases operational costs, and gas-induced temperature gradients can alter droplet freezing behavior. Ultrasonic atomizers generate highly uniform droplets and allow broad particle size tuning; however, they are limited to low-viscosity Newtonian fluids. Piezoelectric droplet-stream generators provide precise control over droplet formation and particle characteristics but may induce coalescence and expose biomacromolecules to mechanical stress [214]. In spray-freezing processes tailored for pulmonary applications, process parameters such as stirring intensity, type and temperature of liquid cryogen, and freezing rate critically influence the microstructure and porosity of frozen droplets, thereby determining the respirable fraction of the final product. Subsequent freeze-drying conditions, including time, chamber pressure, shelf temperature, and drying gas flow rate, affect the preservation of APIs, the stability of excipients, and the overall aerodynamic behavior of the inhaling powder. Optimizing these interrelated parameters ensures the production of particles with suitable size distribution, low density, and high dispersibility, which are essential for efficient lung deposition and therapeutic efficacy.

Brunaugh et al. (2019) [215] investigated the influence of dispersion device design on the aerodynamic performance of spray freeze-dried lysozyme powders, using lysozyme as a model protein. The formulations were evaluated with two DPI systems differing in airflow resistance. The HandiHaler (medium resistance) and the RS01 (high resistance) devices. The resulting particles exhibited an agglomerated morphology, which was strongly influenced by the solid content of the feed solution (1–10%). When dispersed using the HandiHaler, the formulations achieved FPF ranging from 23% to 36%, with MMAD between 6 µm and 13 µm and GSD of 1.3–1.5. In contrast, delivery via the RS01 device yielded higher aerosolization efficiency, with FPF values of 32–52%, smaller MMADs of 3–6 µm, and GSDs between 1.3 and 1.4. These findings highlight the critical role of both formulation composition and device resistance in determining the aerosol performance of spray freeze-dried protein powders intended for pulmonary administration.

A similar trend has been reported for other biologics processes by SFD [216,217,218,219,220]. For example, Emami et al. (2019) [216] studied the development of amino acid-stabilized adalimumab microparticles. SFD produced spherical, highly porous particles (8–12 µm), with SEM showing that excipient type had minimal impact on overall morphology, an observation consistent with the agglomerated yet porous structures described by Brunaugh et al. (2019) [215] for lysozyme powders. The inclusion of amino acids such as leucine, phenylalanine, glycine, or arginine preserved both the physicochemical integrity and biological activity of adalimumab during processing and accelerated storage (3 months). Aerodynamic performance varies depending on the amino acid, with FPF from 25% to 67%, and formulations containing leucine or phenylalanine achieving the highest respirable fractions. These findings parallel the dependence of aerosolization efficiency on formulation parameters seen in the lysozyme study, reinforcing that SFD can reliably generate protein-based particles with suitable aerodynamic behavior for pulmonary delivery when excipient selection and formulation conditions are optimized.

Supercritical fluid (SCF) technologies, particularly the supercritical anti-solvent (SAS), supercritical fluid-assisted spray-drying (SA-SD), solution-enhanced dispersion by supercritical fluids (SEDS), and rapid expansion of supercritical solutions (RESS) processes, have emerged as powerful particle-engineering platforms for producing respirable drug powders with tightly controlled size, morphology, and polymorphism. In these systems, supercritical CO_2_ acts as a tunable solvent, anti-solvent, or co-processing medium that enables rapid mass transfer and near-instantaneous supersaturation, yielding micron- and submicron-sized particles optimized for deep-lung deposition (Figure 6D) [186,221,222]. Carbon dioxide (CO_2_) is the most widely employed supercritical fluid in pharmaceutical applications due to its accessible critical point (31.1 °C, 73.8 bar), non-toxicity, non-flammability, and complete removal by simple depressurization. Upon depressurization below the critical point, supercritical CO_2_ instantaneously reverts to a gaseous state at atmospheric conditions, leaving absolutely no solvent residues in the final product. This eliminates the need for residual solvent testing required for organic solvents (ICH Q3C guidelines classify CO_2_ as Class 5—lowest risk) and represents a significant regulatory advantage, particularly for pulmonary formulations where residual solvent toxicity poses concerns for respiratory tissues [223,224].

Supercritical-fluid processes (SEDS/SAS/RESS and related scfCO_2_-assisted spray-drying methods) routinely produce geometric particle sizes in the submicron-to-single-digit-micrometer range (typically ~0.5–5 µm), with morphologies ranging from dense/spherical to highly porous or “nanocluster” agglomerates that reduce tapped density and lower aerodynamic diameter; these low-density porous structures produce MMADs in the respirable window (~1–5 µm) and substantially narrower APSD than many conventionally milled powders, leading to improved emitted dose and FPF [221,224]. Reported FPFs vary by drug/formulation/device, but studies commonly report improvements from <40% (milled) to ~40–80% (SCF-engineered) depending on excipient content and device. For example, SEDS-engineered budesonide powders showed higher ED (70–80%) and improved FPF (58–69%) versus micronized (36–60%) and commercial comparators (13–31%). SED-engineered MMAD showed values of 3.4–4.5 µm, micronized 3.6–6.8 µm, and commercial comparators < 8.3 µm [225]. Yongda Sun [226] evaluated the carrier-free inhaled dry powder of budesonide tailored by SFD design, showing significantly improved aerodynamic performance compared with milled powder and commercial Pulmicort^®^. The SCF-formulation achieved an FPF of 22–32%, comparable to the marketed Pulmicort^®^ powder at 28% and 7% for milled powder. The MMAD values for formulation were similar, around 4.6–4.9 μm, while the milled powder had an MMAD of 4.6 μm and the marketed Pulmicort^®^ 3 μm. The enhanced aerodynamic performance of the SCF powders was attributed to their lower bulk density, non-spherical morphology, and higher electrostatic charges, which reduced particle agglomeration and improved lung deposition. Budesonide also has been evaluated for respiratory delivery using nebulizers engineered by SCF comparing using jet or vibrating mesh nebulizer system. The formulation showed that using jet nebulizer system an FPF of 9–14%, MMAD of 6–7 µm and GSD of ~1.8, using vibrating mesh the FPF was 32–53%, with MMAD of 2.4–3.5 µm and GSD of 1.9–2.4. These results indicate the potential of supercritical fluid processing technologies in producing an alternative preparation for vibrating mesh nebulizer applications [227]. On the other hand, SCF-assisted spray-drying and SASD show that the parameters could be optimized using DoE/QbD (Design of Experiments/Quality by Design) to tune feed, nozzle, and CO_2_ ratios to obtain spherical or wrinkled composite particles. The chitosan case studied by Cabral et al. (2016) [228] showed aerodynamic diameters (2.1–2.7 µm) and FPFs (38–70%) suitable for DPIs with MMAD of 1.1–2.2 µm and GSD of 2.7–3.3 [228].

Achieving optimal particle size distributions for pulmonary delivery requires sophisticated particle engineering and formulation strategies. Particles must be small enough for respiratory deposition (1–5 μm aerodynamic diameter) yet large enough for physical stability and handling. Balancing these competing requirements, while maintaining drug stability, flowability, and dispersibility, represents a significant formulation challenge. Additionally, many drugs exhibit poor compatibility with conventional carrier particles (typically lactose), leading to inconsistent aerosolization performance.

Each manufacturing technique offers distinct advantages and challenges concerning scalability, process control, and economic considerations. Key challenges include preserving physical stability during processing and storage, achieving consistent batch-to-batch reproducibility, and ensuring scalable, cost-efficient production. Progress in process analytical technologies and deeper insights into structure-property relationships will be essential for refining particle engineering strategies, ultimately enhancing the performance and reliability of pulmonary drug delivery systems [229,230].

Advanced manufacturing enables production of sophisticated nanoparticulate systems offering enhanced antimicrobial delivery.

**Table 3:** Key manufacturing technologies for manufacturing inhalable formulations for infectious respiratory diseases.

Manufacturing Technology	Advantages (+) vs. Limitations (−)	Particle Morphology	Typical Aerodynamic Performance		Ref
			FPF (%)	MMAD (µm)	
Jet milling	(+) Solvent-free, simple, preserves crystallinity. (−) Broad particle size distribution, high cohesiveness requires carrier/force control agents.	Irregular, crystalline, rough-surfaced microparticles	20–40	2–7	[190,231]
Spray drying	(+) High tunability of density/porosity enables “Trojan” nano-in-micro systems. (−) Thermal/shear stress on biologics; hygroscopic powders often need protection.	Spherical, corrugated, or donut-shaped (hollow)	40–80	1–5	[232,233]
Spray freeze drying	(+) Excellent dispersibility due to low density; preserves the bioactivity of proteins. (−) High energy cost; fragile particles are difficult to fill in capsules.	Highly porous, light, fluffy agglomerates	50–70	3–10 (geometric), <5 (aerodynamic)	[234,235,236]
Supercritical fluid technology	(+) Single-step, solvent-free (or low residue), narrow size distribution. (−) High equipment cost; complex scale-up at the industrial level.	Smooth spherical or nanostructured particles	40–75	1–5	[221,237,238,239]

## 8. Nano- and Microparticulate Drug Delivery Systems

Nano- and microparticulate drug delivery systems have emerged as promising strategies for lung deposition, offering enhanced therapeutic outcomes for various pulmonary diseases such as COPD, lung cancer, tuberculosis, and emerging infections like COVID-19 [57,240,241]. These systems utilize advanced technologies to improve drug solubility, target specific lung sites, and minimize systemic side effects (See Table 4). The development of these delivery systems involves a comprehensive understanding of drug types, formulation techniques, clinical outcomes, and regulatory challenges.

Several techniques are employed to produce particles with tunable morphologies, enhancing lung deposition and bioavailability [242,243]. Nanocarriers have shown significant progress in clinical development, with some formulations already commercially available, offering targeted deposition and controlled release in the tracheobronchial tree [57]. The evolution from conventional inhalable formulations to sophisticated nanocarriers, including liposomes, polymeric nanoparticles, and lipid-based systems, has enhanced drug stability, bioavailability, and controlled release profiles [244].

Polymeric nanoparticles (Poly(lactic-co-glycolic acid) (PLGA), chitosan, Poly(lactic acid) (PLA), and other biodegradable polyesters) and lipid carriers (liposomes, solid lipid nanoparticles (SLNs), nanostructured lipid carriers (NLCs)) are widely used for pulmonary delivery with formulation-dependent sizes, drug loads, and aerosol metrics; clinical translation is limited but several liposomal systems reached clinical evaluation [245,246,247]. For example, PLGA is an FDA-approved biodegradable copolymer with tunable degradation rates (days to months) based on lactide: glycolide ratio and molecular weight. Degrades via hydrolysis to lactic acid and glycolic acid, both endogenous metabolites. Chitosan is a natural cationic polysaccharide with mucoadhesive properties, permeation enhancement capability, and inherent antimicrobial activity. Biocompatible and biodegradable via lysozyme-mediated hydrolysis.

Polymeric nanoparticles (NPs) and liposomes can be engineered for receptor-mediated uptake or to avoid phagocytosis by PEGylation. The ability to control cellular uptake enables targeting specific pulmonary cell populations: alveolar macrophages for infectious disease therapy, epithelial cells for cystic fibrosis gene therapy, or tumor cells for lung cancer treatment [247]. Liposomes are self-assembling vesicular structures composed of phospholipid bilayers enclosing aqueous compartments, enabling encapsulation of both hydrophilic (in aqueous core) and hydrophobic (in lipid bilayer) drugs. For pulmonary delivery, liposomal formulations must be converted to dry powder or nebulizable suspension formats while maintaining vesicle integrity [248]. Several liposomal inhalation formulations have entered clinical evaluation for lung fungal infections, cystic fibrosis, and lung cancer; however, only a small number of inhalable nanoformulations have reached regulatory approval to date. The field reports major translational and device-compatibility challenges [125,249]. For example, liposomal amikacin for inhalation (Arikayce^®^) is approved for Mycobacterium avium complex lung disease, demonstrating proof-of-concept for liposomal inhalation therapy [250,251].

SLNs consist of a solid lipid matrix stabilized by surfactants. NLCs, a second-generation formulation, incorporate liquid lipids into this matrix, resulting in a less ordered crystalline structure that provides higher drug loading and reduces drug expulsion during storage. For pulmonary delivery, siRNA can be encapsulated within cationic SLNs and processed via thin-film freeze-drying into an inhalable dry powder. This method maintains nanoparticle integrity and aerosol performance, enabling applications in pulmonary gene silencing [252]. Proliposomes are dry powder formulations that spontaneously form liposomes upon contact with aqueous media (e.g., lung lining fluid). This approach circumvents stability issues of preformed liposomes while enabling dry powder inhaler delivery [253].

Inhaled antibiotics, including tobramycin or colistin, azithromycin (AZM), and levofloxacin, can be directly administered to treat bacterial infections such as *Pseudomonas aeruginosa* in cystic fibrosis, improving therapeutic efficacy while minimizing systemic side effects [254,255]. Liposomal-packaged formulations are designed to penetrate bacterial biofilms and deliver therapeutic doses to infected lung cells, thereby reducing systemic side effects [256]. Inhalable AZM-microparticles have also shown anti-inflammatory effects in macrophages [257]. Fungal infections in the pulmonary system, such as those caused by *Aspergillus* or *Candida* species, are treated with antifungal agents such as voriconazole, amphotericin B, and itraconazole. These antifungals inhibit ergosterol synthesis in fungal cell membranes or form pores, leading to cell death. Parenteral liposomal formulations of amphotericin B are used for nebulization, offering targeted delivery with reduced nephrotoxicity [142,258,259]. Antiviral agents have been repurposed for COVID-19 and other viral respiratory diseases. Remdesivir, an antiviral nucleoside analog, disrupts viral RNA replication and has shown efficacy against SARS-CoV-2 [260]. Inhaled sulphated polysaccharides have demonstrated broad-spectrum antiviral activity by inhibiting viral attachment and entry into host cells, which is crucial for preventing infection progression [261,262]. While polysaccharide sulphates show potential as antiviral agents, alternative strategies, such as using pulmonary surfactant lipids, are also being explored. These lipids have shown efficacy in modulating immune responses and reducing viral replication in respiratory infections, including SARS-CoV-2, highlighting the diverse strategies being investigated for combating respiratory viruses [79,263].

Inhaled siRNA therapeutics must overcome nuclease degradation in airway lining fluid, mucociliary clearance, limited epithelial permeability, and endosomal entrapment. ALN-RSV01, targeting RSV nucleocapsid, demonstrated favorable safety and tolerability in a Phase IIa trial of lung transplant recipients with RSV infection, with significantly lower cumulative symptom scores and reduced incidence of new/progressive bronchiolitis obliterans syndrome at Day 90 (6.3% vs. 50%) compared to placebo. The subsequent Phase IIb trial in lung transplant patients, however, did not meet its primary endpoint of RSV viral load reduction, though secondary clinical analyses suggested potential benefit with early intervention. These findings highlight critical challenges in timing, dosing, and patient selection for prophylactic RSV RNAi therapeutics [264,265]. Current delivery strategies include lipid nanoparticles with ionizable lipids for endosomal escape, polymeric carriers (PEI, chitosan), and cell-penetrating peptide conjugates. For respiratory infections, siRNA targets under investigation include SARS-CoV-2 sequences (spike, RdRp, nucleocapsid), host entry factors (ACE2, TMPRSS2), and pro-inflammatory mediators. Chemical modifications (2′-O-methyl, 2′-fluoro, phosphorothioate) enhance stability while maintaining silencing activity [266].

Airway diseases often coexist with other conditions, such as rheumatoid arthritis (RA) and hematologic malignancies, complicating their management. In RA, pulmonary involvement is a common extraarticular manifestation, and airway disease can significantly impact patient outcomes [267]. Monoclonal antibodies represent a novel therapeutic approach for severe chronic airway diseases, including asthma and chronic rhinosinusitis. These biologics target specific inflammatory pathways, reducing inflammation and improving disease control [268].

The specific problem addressed is the challenge of achieving efficient and reproducible lung deposition of nano- and microparticulate drug formulations while overcoming physiological barriers such as mucociliary clearance, macrophage phagocytosis, and heterogeneous lung microenvironments [269,270,271]. Although numerous nanoparticle-based inhalation systems have been developed, only a few have progressed to clinical approval, revealing a translational gap between bench research and clinical application. Furthermore, regulatory challenges and safety concerns related to nanotoxicology and long-term pulmonary effects complicate clinical translation. Failure to address these gaps limits therapeutic efficacy and patient outcomes in respiratory disease management [57,241,272].

Inhaled biologics represent an emerging frontier for respiratory diseases. Dupilumab (Dupixent^®^), an anti-IL-4Rα monoclonal antibody, received FDA approval in September 2024 for COPD with eosinophilic phenotype (≥300 cells/μL) based on BOREAS and NOTUS Phase III trials demonstrating 30–34% reduction in exacerbations and 80–160 mL FEV_1_ improvement versus placebo [273]. While currently administered subcutaneously, these results validate targeting inflammatory pathways in respiratory infections complicated by Type 2 inflammation. Developing inhaled biologic formulations faces challenges, including protein stability during aerosolization, adequate lung residence time, and specialized delivery devices. Strategies under investigation include spray drying with stabilizing excipients (trehalose, leucine), mesh nebulizers operating at reduced shear, and antibody fragment formats (Fab, nanobodies) better suited for pulmonary delivery [274].

Clinical translation of inhaled anti-infectives has yielded important efficacy data from Phase II/III trials. The RESPIRE program evaluating ciprofloxacin DPI in non-CF bronchiectasis demonstrated significant bacterial load reduction (−3.62 log_10_ CFU/g vs. −0.27 log_10_ CFU/g) in Phase II, though Phase III results showed trends toward benefit without reaching statistical significance, partly due to lower-than-expected exacerbation rates [275]. The ORBIT trials of liposomal ciprofloxacin showed divergent outcomes (ORBIT-4 positive, ORBIT-3 negative), highlighting patient selection challenges [276]. Amikacin liposome inhalation suspension (ALIS/Arikayce^®^) achieved FDA approval for refractory MAC lung disease based on CONVERT trial data showing 29% vs. 9% sputum conversion [277]. For antifungals, PC945, a novel inhaled triazole, demonstrated proof-of-concept efficacy in treatment-refractory invasive aspergillosis [278]. These trials collectively demonstrate that inhaled anti-infectives can achieve clinically meaningful outcomes when appropriately matched to patient populations.

Emerging 3D printing technologies promise personalized anti-infective formulations tailored to individual patient needs.

**Table 4:** (**A**) Antibacterial Formulations for Pulmonary Delivery. (**B**) Antifungal Formulations for Pulmonary Delivery. (**C**) Antiviral Formulations for Pulmonary Delivery. (**D**) Formulations for Pulmonary Delivery of Other Therapeutic Agents. Key: Active Pharmaceutical Ingredient (API), No Reported (NR).

(**A**)									
**API**	**Manufacturing Method**	**Inhalation Dispositive**	**Inhalation Technique**	**Application**	**Type of Particle**	**Formulation Properties**	**Aerodynamic Properties**		**Ref**
							**MMAD (µm)**	**FPF (%)**	
**Azithromycin**	Spray drying	DPI	NGI	Bacterial infections	Microparticles	Well-defined spheres	2.7 ± 0.0	65.4 ± 5.1	[212]
**Tobramycin**	Spray freeze-drying	DPI	NGI	Cystic fibrosis	Microparticles	Loose and porous structure	1.3 ± 0.1	83.3 ± 3.9	[279]
**Rifabutin (RFB) and** **isoniazid (INH)**	Spray drying	DPI	ACI	Tuberculosis	Microparticles	Irregular and acquired corrugated surfaces	3.6 ± 0.3 (RFB)/ 3.9 ± 0.0 (INH)	38.1 ± 1.8 (RFB)/ 38.0 ± 1.6 (INH)	[280]
**Cyclodextrin/Ibuprofen**	Nano-spray-drying	DPI	ACI	Cystic fibrosis treatment	Microparticles	Slightly wrinkled surface	3.8–5.2	15.1–51.0	[281]
**Ciprofloxacin**	Wet milling/spray drying	DPI	ACI	Cystic fibrosis	Nano-in-Microparticles	Spherical shape	3.2–3.7	36.5–41.4	[282]
**Colistin**	Freeze-drying	MDI	ACI	Bacterial infections	Nanoparticles	Smooth surfaces	3.3 ± 1.0	61.1 ± 2.0	[283]
**Colistin**	Spray drying	DPI	NGI	Bacterial infections	Microparticles	Smooth surface/irregular dimpled surface	2.7 ± 0.1	59.5 ± 5.4	[284]
**Levofloxacin**	Solvent evaporation	DPI	ACI	Tuberculosis	Microspheres	Spherical shape	2.1 ± 1.2	75.4 ± 1.4	[285]
**Amikacin liposome**	Microscale flow method	Lamira nebulizer	NGI	Pulmonary nontuberculous mycobacteria	Lipid microparticles	Complete shape of a lipid vesicle	4.8–5.0	50.3–53.5	[286]
(**B**)									
**API**	**Manufacturing Method**	**Inhalation Dispositive**	**Inhalation Technique**	**Application**	**Type of Particle**	**Formulation Properties**	**Aerodynamic Properties**		**Ref**
							**MMAD (µm)**	**FPF (%)**	
**Itraconazole**	Wet milling/co-spray drying	DPI	NGI	Pulmonary aspergillosis	Nano-in-Microparticles	Irregular shapes with aggregated forms and undulations in the particle structure	2.5–3.2	64.6–89.9	[287]
**Amphotericin B (AmB) and Itraconazole (ITR)**	Spray drying	DPI	NGI	Fungal pulmonary infections	Microparticles	Irregular non-smooth collapsed surface	~6.0 (AmB)/ ~3.0 (ITR)	67.0–91.0 (AmB) 25.0–42.0 (ITR)	[258]
**Amphotericin B**	Spray drying	DPI	ACI	Allergic Bronchopulmonary Aspergillosis	Nanostuctured Lipid Carriers	Spherical and uniform shape	3.9–4.7	44.5–49.3	[288]
**Posaconazole**	Wet medium milling/spray-drying	DPI	NGI	Invasive pulmonary aspergillosis	Nanocrystals-agglomerated	Dimpled and hollow shape	2.3–3.3	62.9–78.6	[289]
**Voriconazole**	Thin film freezing	DPI	NGI	*Aspergillus species*	Crystalline and amorphous microparticles	Aggregate particles	2.7–5.2	19.6–46.5	[290]
**Voriconazole**	Spray drying	DPI	NGI	Invasive pulmonary aspergillosis	Microparticles	Irregular with crystalline appearance/spherical appearance and smooth surface	6.1 ± 0.2/3.7 ± 0.1	20.9 ± 2.0/43.6 ± 0.1	[291]
**Itraconazole**	Anti-solvent precipitation/wet milling	DPI	ACI	allergic bronchopulmonary aspergillosis and invasive aspergillosis	Nanoclusters	NR	1.2 ± 0.1	91.8 ± 1.2	[292]
(**C**)									
**API**	**Manufacturing Method**	**Inhalation Dispositive**	**Inhalation Technique**	**Application**	**Type of Particle**	**Formulation Properties**	**Aerodynamic Properties**		**Ref**
							**MMAD (µm)**	**FPF (%)**	
**Heparin sodium**	Jet milling	DPI	NGI	Viruses such as COVID-19	Microparticles	Particle agglomerations	2.6–2.9	61.5–76.8	[192]
***Fluc* mRNA**	Thin-film hydration method	Vibrating Mesh Nebulizer	NGI	Viruses such as COVID-19	Lipid nanoparticles	Complete shape of a lipid vesicle	3.7–4.4	54.0–61.1	[293]
**Camostat mesylate**	Thin-film hydration method	Jet nebulizer	ACI	SARS-CoV-2 Infection	Nanoliposomes	NR	4.2 ± 0.1	42.0 ± 6.9	[294]
**Tamibarotene**	Spray freeze drying/spray drying	DPI	NGI	Influenza and SARS-CoV-2	Microparticles	Spherical structures	NR	10.0–44.0	[295]
**Heparin sodium/AZM**	Spray drying	DPI	NGI	SARS-CoV-2 Infection	Microparticles	Corrugated appearance	2.7–4.4	31.5–54.1	[257]
**Favipiravir**	Rotary evaporation/spray drying	DPI	NGI	SARS-CoV-2	Cocrystal	Dimpled spheres	2.9–4.9	26.7–79.3	[296]
**Zanamivir**	Spray drying	DPI	NGI	Viruses such as influenza (A and B)	Microparticles	Corrugated appearance	2.2–2.3	56.3–58.8	[297]
**Remdesivir**	Film hydration method/freeze drying	Nebulizer	NGI	SARS-CoV-2	Liposomes	Spherical morphology	4.1 ± 0.1/3.6 ± 0.1	56.9 ± 0.4/64.3 ± 2.3	[298]
(**D**)									
**API**	**Manufacturing Method**	**Inhalation Dispositive**	**Inhalation Technique**	**Application**	**Type of Particle**	**Formulation Properties**	**Aerodynamic Properties**		**Ref**
							**MMAD (µm)**	**FPF (%)**	
**Chitosan**	Supercritical CO_2_-assisted atomization	Suitable for DPI	–	Lung cancer treatment	Nano-in-microparticles	Well-defined spheres	1.0–2.0	NR	[299]
**Atorvastatin**	Spray drying	DPI	ACI	pulmonary artery atherosclerosis	Nanoemulsion-loaded microspheres	Irregular morphology	1.0–1.5	45.0–54.0	[300]
**Rivaroxaban**	Bead milling/jet milling	DPI	NGI	Pulmonary embolism	Microparticles	Irregular shapes and sizes	6.5 ± 1.3	45.5 ± 4.9	[301]
**Budesonide**	Spray drying	DPI	NGI	Asthma	Microparticles	Irregular wrinkled structure	3.4–3.7	43.0–47.0	[302]
**Cinaciguat**	Single emulsification	DPI	NGI	Pulmonary hypertension	Microparticles	Porous	4.8–6.1	19.8–36.0	[303]
**Salbutamol sulfate**	Spray drying	DPI	NGI	Asthma, COPD	Microparticles	Spherical shape, dimpled and corrugated surface	4.2 ± 0.1	32.8 ± 0.4	[304]
**Nintedanib**	Antisolvent precipitation/probe ultrasonication	Vibrating Mesh Nebulizer	MSLI	Idiopathic pulmonary fibrosis	Nanocrystals	Irregular shapes	~4.3~4.7	~51.0~54.0	[305]
**Docosahexaenoic acid/S-nitrosothiol**	Synthesizing DHA-SON/isolating macrophage cell membranes/extruding them to prepare RCM	Nebulizer cup	NGI	Ferroptosis-radiotherapy in lung cancer	Nanoformulation	Spherical morphology with a discontinuous membrane layer	3.4 ± 0.1	77.8 ± 4.2	[306]
**Celastrol**	spontaneous low-energy method (mixing specific components in optimized ratios)	Nebulizer cup	NGI	Non-small cell lung cancer	Nanoemulsion	Spherical morphology	4.8 ± 0.2	70.7 ± 5.2	[307]

## 9. Application of 3D Printing Technologies in Pulmonary Drug Delivery

Conventional DPIs, MDIs, and nebulizers exhibit significant inter-device and intra-device variability in aerosol performance, leading to inconsistent lung deposition and therapeutic response [308]. Device resistance, internal geometry, and mouthpiece design profoundly influence patient inhalation flow rates and aerosol generation efficiency, yet these parameters are rarely optimized for individual patients. Furthermore, many patients, particularly pediatric and geriatric populations, struggle with proper device technique, resulting in suboptimal drug delivery and poor disease control [309]. Substantial inter-patient variability in lung anatomy, inhalation capacity, disease state, and coordination ability creates a mismatch between standardized devices and individual patient needs. Pediatric patients require lower doses and device resistances compatible with reduced inhalation flows, while geriatric patients may have compromised coordination and inspiratory capacity. Disease-induced changes in airway geometry and mucus properties further complicate optimal drug delivery. Current one-size-fits-all approaches cannot adequately address this heterogeneity.

Traditional manufacturing methods impose high capital costs for tooling and equipment, creating economic barriers to developing devices for rare diseases or small patient populations. Design iterations require expensive retooling, slowing innovation cycles. Batch manufacturing requires large inventory holdings and complex supply chains, increasing costs and limiting flexibility. These constraints are particularly problematic for personalized medicine applications where small-batch or even individual-patient production may be required [37].

Three-dimensional printing, also termed additive manufacturing (AM), offers transformative capabilities that directly address these limitations. AM represents a fundamentally different manufacturing paradigm compared to traditional subtractive (machining) or formative (molding) processes. In AM, objects are constructed through sequential deposition and consolidation of material layers based on digital three-dimensional models, typically generated using computer-aided design (CAD) software or derived from medical imaging data. Three-dimensional printing enables: (i) fabrication of complex internal geometries impossible to achieve through conventional manufacturing, including intricate channel networks, baffles, and flow-conditioning elements; (ii) rapid prototyping and design iteration without tooling costs, accelerating development timelines from years to months; (iii) on-demand manufacturing enabling personalized device geometries based on individual patient anatomy; (iv) integration of multiple materials and functional components within single manufacturing processes; and (v) distributed manufacturing models that could enable point-of-care production in hospital or pharmacy settings [310,311,312,313,314,315,316]. However, realizing this potential requires overcoming significant technical, regulatory, and translational barriers.

**Figure 7:**
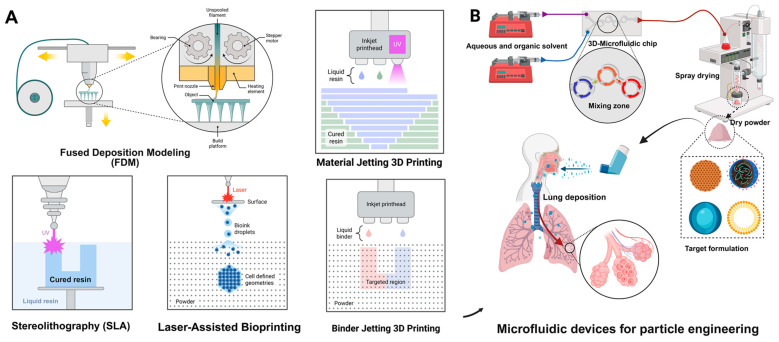
Applications of 3D printing in pulmonary drug delivery. Key: (**A**) Overview of 3D printing technologies applicable to pharmaceutical development: fused deposition modeling (FDM), stereolithography (SLA), laser-assisted bioprinted, material jetting, binder jetting, and (**B**) microfluidic devices for particle engineering for tuning lung deposition (examples of 3D-printed constructs for inhalation research: personalized dose geometries, controlled-release matrices, and device prototypes).

The general 3D printing workflow comprises several sequential steps: (i) digital design creation or acquisition (CAD modeling or medical image segmentation), (ii) conversion to machine-readable format (typically STL files), (iii) slicing and toolpath generation (dividing the model into layers and defining deposition strategies), (iv) physical fabrication through layer-by-layer deposition, (v) post-processing (support removal, curing, surface finishing), and (vi) quality control and characterization. Each step introduces potential sources of variability and requires careful optimization to achieve consistent, high-quality outputs suitable for pharmaceutical applications. Multiple distinct 3D printing technologies have been developed, each based on different physical principles for material deposition and consolidation. For pharmaceutical applications, five primary technologies have received substantial research attention: fused deposition modeling (FDM), selective laser sintering (SLS), stereolithography (SLA), inkjet/material jetting, and binder jetting (Figure 7A). These technologies differ fundamentally in their material compatibility, resolution capabilities, thermal exposure, surface finish characteristics, and suitability for different pharmaceutical applications [311,316].

The convergence of 3D printing with pulmonary drug delivery represents a strategic opportunity to advance personalized respiratory medicine. Patient-specific device geometries could be designed based on computed tomography (CT) or magnetic resonance imaging (MRI) scans to optimize mouthpiece fit and airflow patterns for individual lung anatomy. Complex internal channel designs could be engineered using computational fluid dynamics (CFD) to maximize powder deagglomeration and fine particle generation. Drug-loaded particles with precisely controlled porosity, surface area, and aerodynamic properties could be fabricated to achieve targeted lung deposition patterns. These capabilities align with the broader trajectory toward precision medicine, where therapeutic interventions are tailored to individual patient characteristics, disease phenotypes, and pharmacogenomic profiles [317,318,319].

Yuan et al. (2025) [320] studied the use of 3D printing technology to optimize the droplet size distribution of personal nebulizers, aiming to enhance the effectiveness of inhalation therapy. The study applied 3D printing to modify the small volume nebulizer nozzle baffle, fabricating 27 self-modified nebulization cups with variations in width (W), depth (D), and insertion distance (S) between the baffle and the nozzle, showing that modifying the nebulizer’s baffle to widths of 3–5 mm and depths of 3–5 mm resulted in the most significant reduction in particle size, with FPF up to 67.2% and MMAD around 3.3–3.8 μm, where statistical analysis confirmed that the width (W) of the baffle was the most significant factor affecting droplet size distribution and FPF.

A notable application involves the development of add-on devices for DPIs, where 3D-printed components with adjustable motor power can regulate fan speed and airflow to enhance drug administration efficiency. These devices have shown particular promise for patients with limited inspiratory capacity, including young children, elderly patients, and those with COPD [321].

Recent advances have demonstrated the integration of 3D-printed microfluidic micromixers with spray drying technology for the deep lung antimicrobial microparticle production (Figure 7B). For example, Anaya et al. (2025) [322] developed a novel continuous manufacturing platform consisting of microfluidic micromixers coupled with spray drying for pulmonary delivery applications, demonstrating effective deep lung deposition at both 30 L/min flow rates (FPF of 57.2% ± 7.4% with MMAD of 4.0 µm ± 1.0 µm) and 60 L/min flow rates (FPF of 47.9 ± 1.3 with MMAD of 4.1 µm ± 0.1 µm), making the treatment effective for a wide range of patients, including children and the elderly, regardless of their breathing strength.

The evidence demonstrates significant potential for 3D printing in pulmonary drug delivery, with established applications in device prototyping and emerging applications in integrated manufacturing systems. However, several limitations must be acknowledged. Current challenges in 3D printing for pulmonary drug delivery include manufacturing tolerances, material limitations, and process scalability. The intricate geometry of microfluidic channels remains challenging for existing 3D printing technologies. Additionally, achieving optimal particle sizes and maintaining drug stability during processing requires careful optimization of printing parameters and post-processing conditions. Additionally, the regulatory pathway for 3D-printed pharmaceutical products remains unclear, potentially limiting clinical translation.

## 10. Future Perspective and Conclusions

Despite the extensive research reviewed here, a significant gap remains between the capability of advanced formulation technologies and clinical reality. Currently, apart from the Tobi Podhaler^®^ (tobramycin inhalation powder) for cystic fibrosis, there are no other DPIs commercially approved specifically for the treatment of respiratory infections. The clinical management of non-cystic fibrosis bronchiectasis, nontuberculous mycobacterial pulmonary disease, invasive pulmonary aspergillosis, and bacterial pneumonia in immunocompromised patients continues to rely predominantly on nebulized formulations that repurpose intravenous solutions, despite their suboptimal aerosol characteristics and lengthy administration times (typically 15–30 min per dose) [205,206,208,232].

Tobramycin inhalation powder demonstrates that a DPI formulation engineered specifically for the lung can match or improve the efficacy of nebulized solution, reduce administration time, and enhance adherence without compromising safety. However, the paucity of follow-on products reveals substantial translational barriers. For invasive fungal infections, no DPI formulations of amphotericin B, voriconazole, posaconazole, or isavuconazole have received regulatory approval despite promising preclinical data demonstrating superior lung tissue concentrations compared to intravenous administration. Current clinical practice relies on nebulization of intravenous amphotericin B (conventional or liposomal formulations) for prophylaxis or adjunctive treatment in high-risk hematology-oncology patients, immunocompromised hosts with COVID-19-associated pulmonary aspergillosis, and lung transplant recipients [323,324,325,326].

Similarly, for nontuberculous mycobacterial pulmonary disease, only one inhaled product (Arikayce^®^, liposomal amikacin inhalation suspension) has received FDA approval, and this is limited to the treatment of refractory *Mycobacterium avium* complex lung disease as part of combination therapy [250].

The convergence of advanced particle engineering, sophisticated manufacturing technologies, digital health integration, and evolving regulatory frameworks positions pulmonary drug delivery at an inflection point. After decades of predominantly laboratory-based research, multiple engineered inhalation formulations are progressing through late-phase clinical development targeting unmet needs in infectious diseases, cancer, and inflammatory conditions. The commercial success of Tobi Podhaler^®^ and Arikayce^®^ validates that rationally designed formulations can achieve regulatory approval and market adoption, providing precedents that reduce risk for follow-on products.

Key technological advances enabling this transition include continuous manufacturing platforms with real-time process monitoring ensuring consistent particle characteristics, surface engineering strategies creating stable, highly dispersible powders suitable for DPI delivery, lipid-based and polymeric nanocarrier systems achieving sustained release and enhanced cellular uptake, three-dimensional printing enabling personalized formulations and device optimization, and smart inhaler technologies addressing the critical challenge of medication adherence. These capabilities, matured through extensive preclinical and early-phase clinical validation, are now positioned for broader clinical implementation.

However, realizing the full potential of advanced pulmonary delivery requires addressing persistent challenges such as streamlining regulatory pathways for complex drug-device combination products and novel delivery systems (particularly nanotechnology-based formulations), developing manufacturing processes scalable to commercial production volumes while maintaining the sophisticated particle characteristics achievable at laboratory scale, demonstrating cost-effectiveness that justifies premium pricing compared to established therapies, and conducting adequately powered clinical trials with endpoints meaningful to patients, clinicians, and payers. Collaborative efforts among academia, industry, regulatory agencies, and patient advocacy groups will prove essential for navigating these challenges.

The future of pulmonary drug delivery for infectious diseases extends beyond incremental improvements in existing formulations to encompass transformative new therapeutic modalities. Inhaled bacteriophages, mRNA-based therapeutics, gene editing technologies, and immunotherapies represent the vanguard of next-generation pulmonary medicines that could fundamentally alter disease management. As antimicrobial resistance escalates globally and novel respiratory pathogens continue to emerge, the lung, representing both the primary site of infection and a readily accessible route for therapeutic intervention, will increasingly serve as the focal point for innovative treatment strategies. The particle engineering principles, manufacturing technologies, and translational frameworks established over the past two decades provide the foundation upon which these future therapies will be built, offering hope for improved outcomes in some of medicine’s most challenging infections.

## Data Availability

Data will be made available upon request.

## Abbreviations

The following abbreviations are used in this manuscript:

RSV	Respiratory syncytial virus
COPD	Chronic Obstructive Pulmonary Disease
ILD	interstitial lung diseases
CPA	Chronic Pulmonary Aspergillosis
ICU	Intensive Care Unit
ARDS	respiratory distress syndrome
pMDIs	pressurized metered-dose inhalers
DPIs	dry powder inhalers
HFA	hydrofluoroalkane
MMAD	Mass median aerodynamic diameter
Da	Particle diameter
Dg	Geometric diameter
FPF	fine particle fraction
GSD	geometric standard deviation
CFD	Computational fluid dynamics
ABPA	allergic bronchopulmonary aspergillosis
NGI	Next Generation Impactor
APSD	aerodynamic particle size distribution
PSD	particle size distribution
ACI	Andersen Cascade Impactor
MSLI	Multi-Stage Liquid Impinger
GTI	Glass Twin Impinger
FSA	Fast Screening Andersen
MOC	micro-orifice collector
SEDS	Solution-Enhanced Dispersion by Supercritical Fluids
AZM	azithromycin
RA	rheumatoid arthritis
AM	Additive manufacturing
CT	Computed tomography
MRI	Magnetic resonance imaging
CAD	Computer-aided design
FDM	Fused deposition modeling
SLS	Selective laser sintering
DA	Aerodynamic diameter
PLGA	Poly(lactic-co-glycolic acid)
PLA	Poly(lactic acid)
SLN	Solid lipid nanoparticles
NLC	Nanostructured lipid carriers
NP	Polymeric nanoparticle
ADPIs	acoustic dry powder inhalers
SLA	stereolithography
LCD	liquid crystal display
nCmP	nanocomposite microparticles
LOC	Lung-on-chip
SCF	Supercritical fluid
SAS	Supercritical anti-solvent
SFD	Spray Freeze Drying
SA-SD	supercritical fluid-assisted spray-drying
RESS	Rapid expansion of supercritical solutions
CO_2_	Carbon dioxide
